# Addendum: Designing microbial consortia with defined social interactions

**DOI:** 10.1038/s41589-024-01560-1

**Published:** 2024-04-22

**Authors:** Wentao Kong, James J. Collins, Ting Lu

**Affiliations:** 1 Department of Bioengineering, University of Illinois at Urbana–Champaign, Urbana, IL USA; 2 Carl R. Woese Institute for Genomic Biology, University of Illinois at Urbana–Champaign, Urbana, IL USA; 3 Institute for Medical Engineering and Science, Department of Biological Engineering, and Synthetic Biology Center, Massachusetts Institute of Technology, Cambridge, MA USA; 4 Broad Institute of MIT and Harvard, Cambridge, MA USA; 5 Wyss Institute for Biologically Inspired Engineering, Harvard University, Boston, MA USA; 6 Department of Physics, University of Illinois at Urbana–Champaign, Urbana, IL USA; 7 Center for Biophysics and Quantitative Biology, University of Illinois at Urbana–Champaign, Urbana, IL USA

**Keywords:** Synthetic biology, Metabolic engineering

Correction to: *Nature Chemical Biology* 10.1038/s41589-018-0091-7, published online 25 June 2018.

In our original article, we reported the development of synthetic microbial communities through social interaction engineering that combines experimental strain construction with mathematical modeling. The experimental results, which constitute the primary contribution of the work, remain accurate. However, we have identified the need for corrections in certain modeling results, including the simulation curves of Figs. 4 and 5 in the main text and the Supplementary Information detailing model construction and related simulation materials. The errors, which are now corrected via this amendment, pertain to inaccurate mathematical descriptions of nutrient reduction and nisin synthesis along with discrete errors in equation description and model simulation. Notably, nutrient reduction was initially described solely in terms of nutrient consumption for cell growth. It has now been changed to represent the net nutrient loss, taking into account both consumption during cell growth and recycling from cell death. Additionally, nisin synthesis, originally described as a one-step event, has been elaborated as a two-step process to provide a more detailed description. Due to the interrelated nature of the modeling, updates have been made to the simulation results of Figs. 4 and 5 and Supplementary Figs. 4c, 6d–f, 8, 9a and 11b, h, i, as well as to associated equations, parameters and text. Despite these updates, the overall modeling framework and strategies remain correct and thus unchanged, and the updated model continues to successfully capture and predict the experimental results. The overall scientific conclusions remain intact and accurate. The revised Figs. 4 and 5 are shown below as Figs. 1 and 2, and the amended Supplementary Information is available in the online version of this amendment.

**Fig. 1:**
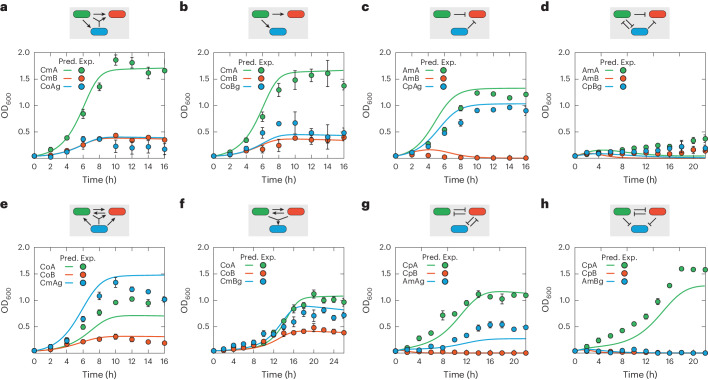
Revised Fig. 4.

**Fig. 2:**
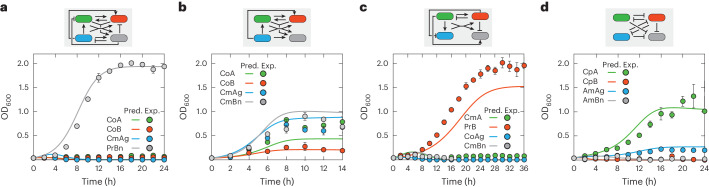
Revised Fig. 5.

### Supplementary information

Revised Supplementary Information

