# Supplementary material for: Addendum: Designing microbial consortia with defined social interactions
Source: Nat Chem Biol. 2024 Apr 22;20(6):789–90. doi: 10.1038/s41589-024-01560-1 (PMC11142905; doi:10.1038/s41589-024-01560-1)
Supplement: Supplementary file 1 — Revised Supplementary Information [file 41589_2024_1560_MOESM1_ESM.pdf]

Addendum: Designing microbial consortia with defined social interactions

In the format provided by the authors and unedited

Supplementary Tables

Supplementary Table 1: Strains and plasmids used in this study

Name	Description	Source or Reference
**Strains**		
Lactococcus lactis NZ9000	Host for all strains in all ecosystems; *nisRK* integrated into chromosome	(1)
NeA	Strain A in neutralism; Contains the plasmids pCCAMβ1 and pleiss-Pcon-gfp; Erm^R, Cm^R	This study
NeB	Strain B in neutralism; Contains the plasmids pCCAMβ1 and pleiss-Pcon-rfp; Erm^R, Cm^R	This study
AmA	Strain A in amensalism; Contains the plasmids pWK6b-RK^- and pleiss-Pcon-gfp; Nisin producer; Erm^R, Cm^R	This study
AmB	Strain B in amensalism; Contains the plasmids pCCAMβ1 and pleiss-Pcon-rfp; Nisin and lcnA sensitive; Erm^R, Cm^R	This study
AmA2	Strain A in amensalism; Contains the plasmids pWK-$lcnA^{wt}$ and pleiss-Pcon-gfp; lcnA producer; Erm^R, Cm^R	This study
CmA	Strain A in commensalism; Contains the plasmids pWK6b-RK^- and pleiss-Pcon-tet-Pcon-gfp; nisin producer; Erm^R, Cm^R; Tet^R	This study
CmB	Strain B in commensalism; Contains the plasmids pWK6-IFEG and pleiss-Pnis-tet-Pcon-rfp; nisin resistant; Erm^R, Cm^R; nisin inducible Tet^R	This study
CmA(Pnis-tet)	Strain A in commensalism; Contains the plasmids pWK6b-RK^- and pleiss-Pnis-tet-Pcon-gfp; nisin producer; Erm^R, Cm^R; self-inducible Tet^R	This study
CoA	Strain A in cooperation; Contains the plasmids pWK6b-PRK^- and pleiss-Pnis-tet-Pcon-gfp; nisin precursor producer; Erm^R, Cm^R; nisin inducible Tet^R	This study
CoB	Strain B in cooperation; Contains the plasmids pWK6-IPFEG and pleiss-Pnis-tet-Pcon-rfp; nisin resistant and nisP producer; Erm^R, Cm^R; nisin inducible Tet^R	This study
CpA	Strain A in competition; Contains the plasmids pWK6-RK^- and pleiss-Pcon-gfp; nisin producer and lcnA sensitive; Erm^R, Cm^R	This study
CpA2	Strain A in competition; Same as AmA; Contains the plasmids pWK6b-RK^- and pleiss-Pcon-gfp; Nisin producer; Erm^R, Cm^R	This study
CpB	Strain B in competition; Contains the plasmids pWK-$lcnA^{5K}$ and pleiss-Pcon-rfp; lcnA producer and nisin sensitive; Erm^R, Cm^R	This study
CpB2	Strain B in competition; Contains the plasmids pWK-$lcnA^{wt}$ and pleiss-Pcon-rfp; lcnA producer and nisin sensitive; Erm^R, Cm^R	This study

Name	Description	Source or Reference
CpB3	Strain B in competition; Contains the plasmids pWK-lcnA^{20k} and pleiss-Pcon-rfp; lcnA producer and nisin sensitive; Erm^{R}, Cm^{R}	This study
PrA	Strain A (prey) in predation; Same as CmA; Contains the plasmids pWK6b-RK^{-} and pleiss-Pcon-tet-Pcon-gfp; nisin producer; Erm^{R}, Cm^{R}; Tet^{R}	This study
PrB	Strain B (predator) in predation; Contains the plasmids pWK6-IFEG-lcnA^{wt} and pleiss-Pnis-tet-Pcon-rfp; lcnA producer and nisin resistant; Erm^{R}, Cm^{R}; nisin inducible Tet^{R}	This study
PrA (Pnis-tet)	Strain A (prey) in predation; Same as CmA (Pnis-tet); Contains the plasmids pWK6b- RK^{-} and pleiss-Pnis-tet-Pcon-gfp; nisin producer; Erm^{R}, Cm^{R}; self-inducible Tet^{R}	This study
CoAg	Third strain in three- and four-strain ecosystem; Constructed by replacing the gfp reporter in CoA with gusA reporter	This study
CoBg	Third strain in three- and four-strain ecosystem; Constructed by replacing the rfp reporter in CoB with gusA reporter	This study
CpAg	Third strain in three- and four-strain ecosystem; Constructed by replacing the gfp reporter in CpA with gusA reporter	This study
CpBg	Third strain in three- and four-strain ecosystem; Constructed by replacing the rfp reporter in CpB with gusA reporter	This study
CmAg	Third strain in three- and four-strain ecosystem; Constructed by replacing the gfp reporter in CmA with gusA reporter	This study
CmBg	Third strain in three- and four-strain ecosystem; Constructed by replacing the rfp reporter in CmB with gusA reporter	This study
AmAg	Third strain in three- and four-strain ecosystem; Constructed by replacing the gfp reporter in AmA with gusA reporter	This study
AmBg	Third strain in three- and four-strain ecosystem; Constructed by replacing the rfp reporter in AmB with gusA reporter	This study
PrBn	Fourth strain in four-strain ecosystem; Constructed by deleting the rfp reporter in PrB ; No reporter	This study
CmBn	Fourth strain in four-strain ecosystem; Constructed by deleting the rfp reporter in CmB; No reporter	This study
AmBn	Fourth strain in four-strain ecosystem; Constructed by deleting the rfp reporter in AmB; No reporter	This study
**Plasmids**		
pleiss:Nuc	*E. coli* and *L. lactis* shuttle vector for cloning reporter and selector; pSH71 origin; PnisA promoter; Cm^{R}	(2)
pCCAMβ1	*E. coli* and *L. lactis* shuttle vector for cloning nisin or lcnA gene cluster; pCC1BAC and pAMβ1 origins; Erm^{R}	(3)
pWK6	Wild-type of nisin gene cluster in pCCAMβ1	(4)

Name	Description	Source or Reference
pWK6b	Reduced nisB RBS strength in pWK6	This study
pWK-lcnA^{wt}	Wild-type of lcnA gene cluster in pCCAMβ1	This study
pWK-lcnA^{5k}	Reorganized lcnA gene cluster and reduced the strength of RBS of lcnA to 5000 AU	This study
pWK-lcnA^{20k}	Reorganized lcnA gene cluster and increased the strength of RBS of lcnA to 20,000 AU	This study
pWK6-RK^{-}	NisRK knock-out in pWK6	This study
pWK6b-RK^{-}	NisRK knock-out in pWK6b	This study
pWK6b-PRK^{-}	NisP and nisRK knockout in pWK6b	This study
pWK6- IFEG	NisA mutation, nisBTC, nisP and nisRK knock-out in pWK6; nisI and nisFEG have their full functions	This study
pWK6- IPFEG	NisA mutation, nisBTC and nisRK knock-out in pWK6; nisI, nisP and nisFEG have their full functions	This study
pWK6-IFEG-lcnA^{wt}	NisA mutation, nisBTC, nisP and nisRK knock-out in pWK6; nisI and nisFEG have their full functions; Wild-type lactococcin A gene cluster was inserted downstream of nisin gene cluster	This study
pleiss-Pcon-gfp	Green fluorescence reporter; Constitutive expression of gfp	This study
pleiss-Pcon-rfp	Red fluorescence reporter; Constitutive expression of rfp	This study
pleiss-Pcon-tet-Pcon-gfp	Green fluorescence reporter and Tet selector; Constitutive expression of gfp and tet^{R}	This study
pleiss-Pnis-tet-Pcon-gfp	Green fluorescence reporter and Tet selector; Constitutive expression of gfp and nisin inducible expression of tet^{R}	This study
pleiss-Pnis-tet-Pcon-rfp	Red fluorescence reporter and Tet selector; Constitutive expression of rfp and nisin inducible expression of tet^{R}	This study
pleiss-Pnis-tet-Pcon-gusA	GusA reporter and Tet selector; Constitutive expression of gusA and nisin inducible expression of tet^{R}	This study
pleiss-Pcon-tet-Pcon-gusA	GusA reporter and Tet selector; Constitutive expression of gusA and tet^{R}	This study
pleiss-Pcon-gusA	GusA reporter; Constitutive expression of gusA	This study
pleiss-Pnis-tet	No reporter; Nisin inducible expression of tet^{R}	This study

Supplementary Table 2: Primers used in this study

Name	Sequence (5'-3')	Description
Primers for plasmids pWK6-RK$^-$ and pWK6b-RK$^-$ (Nisin gene cluster plasmids in CmA, AmA, CpA, CpA2 and PrA)		
nisPaadA	AGACTAAAAATTGATAGATTATATTTCTTCAGAATGAA TGCCTTGCCGTAGAAGAACAGC	Primers for amplification of aadA and nisRK knock-out
nisFaadA	AAAAGTATATAAAACAATTTTCAGAATCTATTCAGAAA CATGCGATAACAAGAAAAAGCC	
nisP3F-seq	GTTTCTGTACGAAGTCAAG	nisRK knock-out verification and sequencing primers
nisF5R-seq	CTCCTAATAAACCACAGAC	
Primers for pWK6-IPFEG (Nisin resistant and nisP$^+$ plasmid in CoB)		
nisP3KnSmF	AGACTAAAAATTGATAGATTATATTTCTTCAGAATGAA TGGGCCTGGTGATGATGGCG	Primers for replacing nisRK with knstrep cassette
nisF5KnSmR	AAGTATATAAAACAATTTTCAGAATCTATTCAGAAACA TCAGAAGAACTCGTCAAGAAGG	
RK5R	AGAATCTATTCAGAAACACATTCATTCTGAAGAAATAT AATCT	Primers for generating a fragment to replace knstrep by counter selection; The fragment contains 3' of nisP and 5' of nisF and was generated by OE-PCR
RK5F	ATTTTACCTGTTACTGGAG	
RK3F	TTTCTTCAGAATGAATGTGTTTCTGAATAGATTCTGAA AATTG	
RK3R	CTTGTAGCACCTGCTTTTC	
BKnSmF	AAATTATTTGAATCTATTTACAAGTATTATAAGAGAAG TTAGGCCTGGTGATGATGGCG	Primers for replacing nisBTC with knstrep
CKnSmR	GACCTCCATAGCACCATGCATCTCTAATGAAACTTGCT TCAGAAGAACTCGTCAAGAAGG	
B5F	TGGAACAGTTACTACTAGC	Primers for generating a fragment to replace knstrep by counter selection; The fragment contains 5' of nisB and 3' of nisC and was generated by OE-PCR
B5R	TCTAATGAAACTTGCTTAACTTCTCTTATAATACTTGTA AAT	
C3F	GTATTATAAGAGAAGTTAAGCAAGTTTCATTAGAGATG CATG	
CRseq	AATCAATACCAAGTTTCCT	
Primers for the nisin resistant plasmid pWK-IFEG in CmB (primers for nisBTC knock-out are same as pWK6-IPFEG)<br>Primers for nisP and nisRK knock-out are also used for construction of pWK6b-PRK$^-$ in CoA		
nisP5knSmF	TATCGTTTGTTCGTTGGGTTTATCAGCAACTGTGCATG GGGGCCTGGTGATGATGGCG	Primers for replacing nisP and nisRK with knstrep
nisF5KnSmR	AAGTATATAAAACAATTTTCAGAATCTATTCAGAAACA TCAGAAGAACTCGTCAAGAAGG	
nisP5F	TCTCTTACTGAAGCATTTGC	Primers for generating a fragment to replace knstrep by counter selection; The fragment contains 5' of nisP and 3' of nisRK and generated by OE-PCR
nisP5R	TCAGAATCTATTCAGAAACACCCATGCACAGTTGCTGA TAAAC	
RK3Fnew	AGCAACTGTGCATGGGTGTTTCTGAATAGATTCTGAAA ATTG	
RK3R	CTTGTAGCACCTGCTTTTC	

Name	Sequence (5'-3')	Description
Primers for the plasmid pWK6-IFEG-lcnA^{wt} in PrB		
nisP5amp	TATCGTTTGTTCGTTGGGTTTATCAGCAACTGTGCATG GGTCTTAGACGTCAGGTGGCAC	Primers for nisP and nisRK knock-out
nisF5amp	AAAAGTATATAAAACAATTTTCAGAATCTATTCAGAAA CAACGCTCAGTGGAACGAAAAC	
BKnSmF	AAATTAATTTATTACACACTTCAAAGGTTGTTTGTTTCG GAAGGCCTGGTGATGATGGCG	nisBTC knock-out primers
CKnSmR	GACCTCCATAGCACCATGCATCTCTAATGAAACTTGCT TCAGAAGAACTCGTCAAGAAGG	
NotnisinF	CACGTTAACCGGGCTGCAT	Primers for amplifying nisin gene cluster (ABTCPRK^{-}) and cloning into NotI site of pWK-lcn^{wt}
arm2R	AGAGTTCATAAACAATCCTGCATGATAACCATCACAA GCGGCCGCAATACGAATCCATC	
Primers for plasmids pWK-lcnA^{5k}and pWK-lcnA^{20K}		
IR	AAGAAGGTTTTTATATTACAGCTCCAAGATCTTTGCTT AATCAATGGCACGTCTCTT	Primers for amplification of lciA
IF	TAACATTTGTTAACGAGTTTTATTTTTATATAATCTATA ATAGATTTATAAAAATAAGGAGATTATTATGAAAAAG AAACAAATAGAATT	
CF	CCATTCTTAAATAAAAAAAGATTAATCAGTAAGTAATA TTATTTTCATTTATC	Primers for amplification of lceA and lcmA
DR	ATAAAAATAAAACTCGTTAACAAATGTTAATAACTCTA CTGATTGCCTCTTCCCATTATT	
P32F	GATAAATGAAAATAATATTACTTACTGATTAATCTTTT TTTATTTAAGAATGG	Primers for amplification of P32 promoter and change TIR of RBS to 5078 AU
P32-5078R	TTCATTTCAAAGGCTATATCCCTTATTTTTTTACCTACC TAGTATAGC	
5078-AF	TAAAAAAATAAGGGATATAGCCTTTGAAATGAAAAAT CAATTAAATT	Primers for amplification of lcnA and change TIR of RBS to 5078 AU
AR	AGATTGCCGAAAATATGCACTCGAGGTCGACTCAATG GTGCAACCCCGAAAC	
P32-19950R	TTCATTTCAAATAGGTCCTCCTTATTTTTTTACCTACCT AGTATAGC	Primer for amplification of P32 promoter and change TIR of RBS to 19950 AU
19950-AF	TAAAAAAATAAGGAGGACCTATTTGAAATGAAAAATC AATTAAATT	Primer for amplification of lcnA and change TIR of RBS to 19950 AU
leissalF	GTCGACCTCGAGTGCATATTTTC	Primers for amplification of origin and cm^{R} in pleiss-Nuc
bglleissR	TCTTGGAGCTGTAATATAAAAACCTTC	
ambnotleissF	ATCCGATGCAAGTGTGTCGCTGTCGACGCGGCCGCTTG TGATGGTTATCATGCAGGATTG	Primers for subcloning of lcnA gene cluster from pleiss-Nuc to pCCAMβ1
ErmnotleissR	CCTGGTTGCAAATTTTGAAAACCGCTACGGATCGCCGA CATCATTGAACATGCTGAAGAG	

Name	Sequence (5’-3’)	Description
Primers for pWK-lcnA^{wt}		
ambnotleissF	ATCCGATGCAAGTGTGTCGCTGTCGACGCGGCCGCTTGTGATGGTTATCATGCAGGATTG	Amplification of lciA
lciAF	GGTTGCACCATTGAGGATTAGTTAAGATATGAAAAAGAAACAAATAGAATT	
lcnAR	ATTTGTTTCTTTTTCATATCTTAACTAATCCTCAATGGTGCAACCCGAAAC	Amplification of lcnA
lcnAF	ATTTATAAAAATAAGGAGATTATTATGAAAAATCAATTAAATTTTAATAT	
RBSlcnAR	ATTAAAATTTAATTGATTTTTCATAATAATCTCCTTATTTTTATAAATCT	Amplification of lceA and lcmA
notlceAF	CCTGGTTGCAAATTTTGAAAACCGCTACGGATCGCAATCAGTAAGTAATATTATTTTCAT	
Primers for reporter and selector plasmids		
Pcongf	GGATCTAGATAACTGGAATAATCAACCAAATAG	Primers for constructing pleiss-pcon-gfp
leissF	GGATCTAGACTCGAGTGCATATTTTCGGCAATCT	
PconrfpF	TAAAAATAAGGAGATTATTATGGTTTCAAAAGGAGAAGAAGAT	rfp primers for constructing pleiss-pcon-rfp
gfprfpR	ATGAATTATACAAATAA TTATTTGTATAATTCATCCATACC	
rfpPconR	TCTCCTTTTGAAACCATAATAATCTCCTTATTTTTATAAATCT	pleiss primers for constructing pleiss-pcon-rfp
rfpgfpF	TGAATTATACAAATAAGTTTGTAACTGCTGCTGGGATT	
leissalF	GTCGACCTCGAGTGCATATTTTC	Primers for construction of pleiss-Pnis-tet-Pcon-gfp or pleiss-Pnis-tet-Pcon-rfp
leisspnisAR	CATTTTGAGTGCCTCCTTATAATTT	
PnistetF	GGAACTACAAAATAAATTATAAGGAGGCACTCAAAATGAATACATCCTATTCACAATCGA	
grptetR	ATCTATTTGGTTGATTATTCCAGTTTTAGAAATCCCTTTGAGAATGTTTA	
tetgrpF	TAAACATTCTCAAAGGGATTTCTAAAACTGGAATAATCAACCAAATAGAT	
leissgrpR	TTGAGAAGATTGCCGAAAATATGCACTCGAGGTCGACGATCTTTGCTTAATCAATGGCAC	
gfpARBSF	CATGGCATGGATGAGTAAAGGAACTACAAAATAAATTATAAG	Primers for construction of pleiss-Pcon-tet-Pcon-gfp
tertetR	GTTGATTTATTACTCATCCATGCCTTAGAAATCCCTTTGAGAATGTTTA	
tetterF	TAAACATTCTCAAAGGGATTTCTAAGGCATGGATGAGTAATAAATCAAC	
ARBSgfpR	CTTATAATTTATTTTGTAGTTCCTTTACTCATCCATGCCATGTGTAAT	
PconR	CATAATAATCTCCTTATTTTTATAAATC	Primers for construction of pleiss-Pcon-gusA, pleiss-Pcon-tet-Pcon-gusA and pleiss-Pnis-tet-Pcon-gusA
GFP3F	ATGGTCCTTCTTGAGTTTGT	
congusA	TCTATAATAGATTTATAAAAATAAGGAGATTATTATGGAATCTGCACTATATC	

**Name**	**Sequence (5’-3’)**	**Description**
gfp3gusA	ATCCCAGCAGCAGTTACAAACTCAAGAAGGACCAT TTAATTTAATTGTTGCCATCTC	
ssDNA oligos		
nisAmut	A*C*AGATACCAAATCCAAGTTAAAATCTTTTGTACTC AGTTTGAGTGGGAGGTTATAATTTATTTTGTAGTTCCTT CGAACGAAATCATTG	Mutation of RBS and start codon of nisA for pWK6-IFEG, pWK6-IPFEG and pWK6-IPFEG-lcnA^{wt}
nisB269	A*A*AACGGTTGAGCTTTAAATGAACTTTTTATCATGTT TTTTTCTACCGTTATTTTTATAAGCTATTTAGCAACCCT AAATAACTTATAAA	Reduce TIR of nisB RBS to 269 AU

The symbol * stands for the nucleotide has been phosphorothioated.

**Symbol**	**Definition**
N_i	Population of strain i
S_n	Signaling molecule nisin
S_p	Signaling molecule prenisin
S_l	Signaling molecule lcnA
F	Nutrient concentration
μ_i	Maximum growth rate of strain i
K_i	Monod parameter of strain i
γ_i	Yield from nutrient for strain i
d_{S_n}	Death rate nisin
K_{S_n}	Nisin half kill parameter
d_{S_l}	Death rate lcnA
K_{S_l}	lcnA half kill parameter
d_t	Death rate Tet
k_s	Strength of nisin-induced tet^R immunity
α	Nisin production rate
K_p	Half production rate nisin
r_b	Basal rate nisin production
r_l	Basal rate lcnA production
k_m	Rate of nisin modification
k_n, k_p	Rate of nisin and prenisin decay
k_l	Rate of lcnA decay
ε_n, ε_l, ε_t	Nutrient recycling factor (nisin, lcnA, Tet)
D	Diffusion of bacteria
D_f	Diffusion of nutrients
D_n	Diffusion of nisin
D_p	Diffusion of prenisin
D_l	Diffusion of lcnA

Supplementary Table 3: Summary of variables and constants used in simulations.

Parameter	Value	Parameter	Value	Parameter	Value
μ_{CmA}	0.98	K_{CmA}	0.70	γ_{CmA}	2.41
μ_{CmB}	0.78	K_{CmB}	0.75	γ_{CmB}	2.22
α_{CmA}	2.00	K_p	8.0	r_b	0.06
$d_{t,CmB}$	0.36	$k_{s,CmB}$	1.11e3	k_n	0.065
ε_t	0.75				

Parameter	Value	Parameter	Value	Parameter	Value
μ_{AmA}	4.1	K_{AmA}	4.9	γ_{AmA}	2.41
μ_{AmB}	1.53	K_{AmB}	1.14	γ_{AmB}	3.0
α_{AmA}	3.0	K_p	8.0	r_b	0.06
$d_{S_n,AmB}$	0.62	$K_{S_n,AmB}$	5.4e-3	k_n	0.065
ε_n	0.75				

Parameter	Value	Parameter	Value	Parameter	Value
μ_{AmA2}	6.1	K_{AmA2}	7.6	γ_{AmA2}	2.55
μ_{AmB}	1.53	K_{AmB}	1.14	γ_{AmB}	3.0
$r_{l,AmA2}$	2.88	$d_{S_l,AmB}$	1.28	$K_{S_l,AmB}$	0.76
k_l	0.115	ε_l	0.75		

Parameter	Value	Parameter	Value	Parameter	Value
μ_{NeA}	1.14	K_{NeA}	0.65	γ_{NeA}	3.1
μ_{NeB}	1.42	K_{NeB}	1.10	γ_{NeB}	3.1

Supplementary Table 4: Parameter values for well-mixed commensalism (top), amensalism nisin (2nd from top), amensalism lcnA (2nd from bottom), and neutralism (bottom) simulations.

Parameter	Value	Parameter	Value	Parameter	Value
μ_{CoA}	0.58	K_{CoA}	0.278	γ_{CoA}	2.70
μ_{CoB}	1.00	K_{CoB}	1.50	γ_{CoB}	2.20
α_{CoA}	2.43	$d_{t,CoA}$	0.40	$k_{s,CoA}$	1.43e3
$k_{m,CoB}$	0.43	$d_{t,CoB}$	0.34	$k_{s,CoB}$	1.47e3
k_n, k_p	0.065	K_p	8.0	r_b	0.06
ε_t	0.75				

Parameter	Value	Parameter	Value	Parameter	Value
μ_{CpA}	1.29	K_{CpA}	1.21	γ_{CpA}	2.02
μ_{CpB}	0.87	K_{CpB}	0.66	γ_{CpB}	2.80
α_{CpA}	3.0	$d_{S_l,CpA}$	1.28	$K_{S_l,CpA}$	0.76
$r_{l,CpB}$	2.88	$d_{S_n,CpB}$	1.97	$K_{S_n,CpB}$	5.0e-3
μ_{CpA2}	0.85	K_{CpA2}	0.47	γ_{CpA2}	2.39
α_{CpA2}	0.080	$d_{S_l,CpA2}$	1.28	$K_{S_l,CpA2}$	0.76
μ_{CpB2}	4.1	K_{CpB2}	5.0	γ_{CpB2}	4.2
$r_{l,CpB2}$	4.5	$d_{S_n,CpB2}$	1.97	$K_{S_n,CpB2}$	5.0e-3
μ_{CpB3}	1.70	K_{CpB3}	1.44	γ_{CpB3}	2.97
$r_{l,CpB3}$	6.0	$d_{S_n,CpB3}$	1.97	$K_{S_n,CpB3}$	5.0e-3
k_n	0.065	K_p	8.0	r_b	0.06
k_l	0.115	ε_n	0.75	ε_l	0.75

Parameter	Value	Parameter	Value	Parameter	Value
μ_{PrA}	0.99	K_{PrA}	0.70	γ_{PrA}	2.22
μ_{PrB}	0.49	K_{PrB}	1.34	γ_{PrB}	1.80
α_{PrA}	2.00	$d_{S_l,PrA}$	1.28	$K_{S_l,PrA}$	0.76
$r_{l,PrB}$	2.00	$d_{t,PrB}$	0.181	$k_{s,PrB}$	2.41e4
k_n	0.065	K_p	8.0	r_b	0.06
k_l	0.115	ε_t	0.75	ε_l	0.75

Supplementary Table 5: Parameter values for well-mixed cooperation (top), competition (middle), and predation (bottom) simulations.

**Strain**	μ	K	γ
$CpAg$	1.02	0.82	2.26
$CpBg$	0.60	0.37	2.90
$CoAg$	0.65	0.50	2.40
$CoBg$	0.58	0.36	2.29
$CmAg$	2.66	3.5	2.33
$CmBg$	0.90	0.90	2.64
$AmAg$	0.70	0.40	2.57
$AmBg$	0.73	0.268	2.95
$AmBn$	1.83	1.44	2.61
$CmBn$	1.74	1.48	2.66
$PrBn$	5.7	10.6	2.00

Supplementary Table 6: Growth parameters for new strains in three- and four-strain ecosystems.

**Parameter**	**Value**	**Parameter**	**Value**	**Parameter**	**Value**
μ_{NeA}	1.14	K_{NeA}	0.65	γ_{NeA}	3.1
μ_{NeB}	1.42	K_{NeB}	1.10	γ_{NeB}	3.1
D_0	5.0e-4	D_f	1.44e-3		

**Parameter**	**Value**	**Parameter**	**Value**	**Parameter**	**Value**
μ_{CoA}	0.58	K_{CoA}	0.278	γ_{CoA}	2.70
μ_{CoB}	1.00	K_{CoB}	1.50	γ_{CoB}	2.20
α_{CoA}	2.43	$d_{t,CoA}$	0.40	$k_{s,CoA}$	1.43e3
$k_{m,CoB}$	0.43	$d_{t,CoB}$	0.34	$k_{s,CoB}$	1.47e3
k_n, k_p	0.265	K_p	8.0	r_b	0.06
ε_t	0.75	D_0	5.0e-4	D_f	1.44e-3
D_n	1.44e-4	D_p	1.44e-4		

**Parameter**	**Value**	**Parameter**	**Value**	**Parameter**	**Value**
μ_{CpA}	1.29	K_{CpA}	1.21	γ_{CpA}	2.02
μ_{CpB}	0.87	K_{CpB}	0.66	γ_{CpB}	2.80
α_{CpA}	3.0	$d_{S_l,CpA}$	1.28	$K_{S_l,CpA}$	0.76
$r_{l,CpB}$	2.88	$d_{S_n,CpB}$	1.97	$K_{S_n,CpB}$	5.0e-3
μ_{CpA2}	0.85	K_{CpA2}	0.47	γ_{CpA2}	2.39
α_{CpA2}	0.080	$d_{S_l,CpA2}$	1.28	$K_{S_l,CpA2}$	0.76
μ_{CpB2}	4.1	K_{CpB2}	5.0	γ_{CpB2}	4.2
$r_{l,CpB2}$	4.5	$d_{S_n,CpB2}$	1.97	$K_{S_n,CpB2}$	5.0e-3
μ_{CpB3}	1.70	K_{CpB3}	1.44	γ_{CpB3}	2.97
$r_{l,CpB3}$	6.0	$d_{S_n,CpB3}$	1.97	$K_{S_n,CpB3}$	5.0e-3
k_n	0.265	K_p	8.0	r_b	0.06
k_l	0.315	ε_n	0.75	ε_l	0.75
D_0	5.0e-4	D_f	1.44e-3	D_n	1.44e-4
D_l	5.76e-3				

Supplementary Table 7: Parameters values for spatial neutralism (top), cooperation (middle), and competition (bottom) simulations.

Supplementary Figures

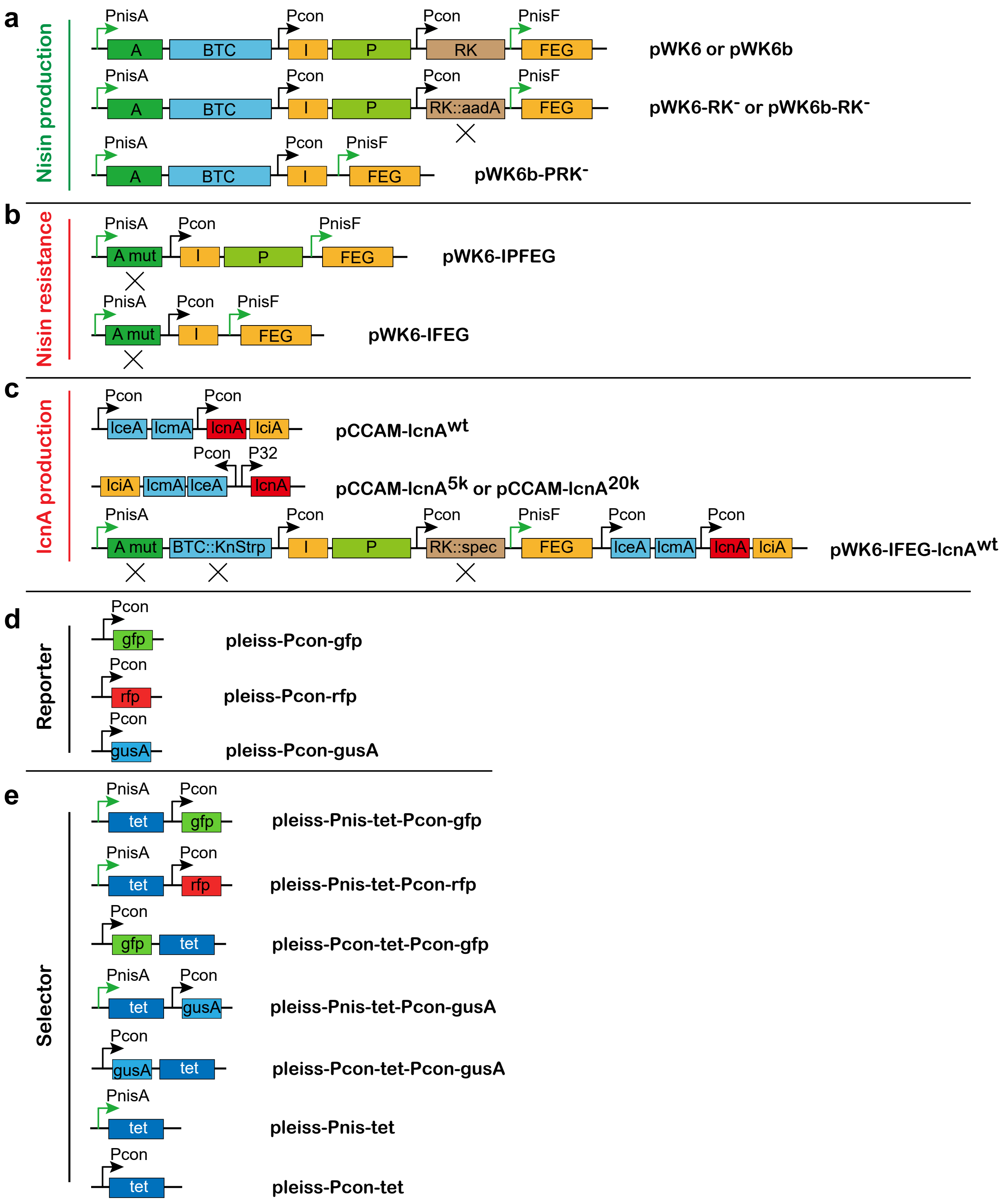

Supplementary Figure 1: Gene organization of the plasmids used in the study.
(**a**) Plasmids for nisin production. (**b**) Plasmids for nisin resistance. (**c**) Plasmids for lcnA production. (**d**) Reporter plasmids. (**e**) Selector plasmids. Cross symbol indicates mutated genes. Strains in each ecosystem contain plasmids with the following combinations: CmA or PrA: pWK6b-RK$^-$ + pleiss-Pcon-tet-Pcon-gfp; CmA(Pnis-tet) or PrA(Pnis-tet): pWK6b-RK$^-$ + pleiss-Pnis-tet-Pcon-gfp; CmB: pWK6-IFEG + pleiss-Pnis-tet-Pcon-gfp; AmA: pWK6b-RK$^-$ + pleiss-Pcon-gfp; AmA2: pCCAM-lcnA^{wt} + pleiss-Pcon-gfp; AmB: pCCAMβ1 + pleiss-Pcon-rfp; NeA: pCCAMβ1 + pleiss-Pcon-gfp; NeB: pCCAMβ1 + pleiss-Pcon-rfp; CpA: pWK6-RK$^-$ + pleiss-Pcon-gfp; CpA2: pWK6b-RK$^-$ + pleiss-Pcon-gfp; CpB: pCCAM-lcnA^{5k} + pleiss-Pcon-rfp; CpB2: pCCAM-lcnA^{wt} + pleiss-Pcon-rfp; CpB3: pCCAM-lcnA^{20k} + pleiss-Pcon-rfp; CoA: pWK6b-PRK$^-$ + pleiss-Pnis-tet-Pcon-gfp; CoB: pWK6-IPFEG + pleiss-Pnis-tet-Pcon-rfp; PrB: pWK6-IFEG-lcnA^{wt} + pleiss-Pnis-tet-Pcon-rfp; CoAg: pWK6b-PRK$^-$ + pleiss-Pnis-tet-Pcon-gusA; CoBg: pWK6-IPFEG + pleiss-Pnis-tet-Pcon-gusA; CpAg: pWK6-RK- + pleiss-Pcon-gusA; CpBg: pCCAM-lcnA^{5k} + pleiss-Pcon-gusA; CmAg: pWK6b-RK$^-$ + pleiss-Pcon-tet-Pcon-gusA; CmBg: pWK6-IFEG + pleiss-Pnis-tet-Pcon-gusA; AmAg: pWK6b-RK$^-$ + pleiss-Pcon-gusA; AmBg: pCCAMβ1+pleiss-Pcon-gusA; AmBn: pCCAMβ1+pleiss; CmBn: pWK6-IFEG + pleiss-Pnis-tet; PrBn: pWK6-IFEG-lcnA^{wt} + pleiss-Pnis-tet.

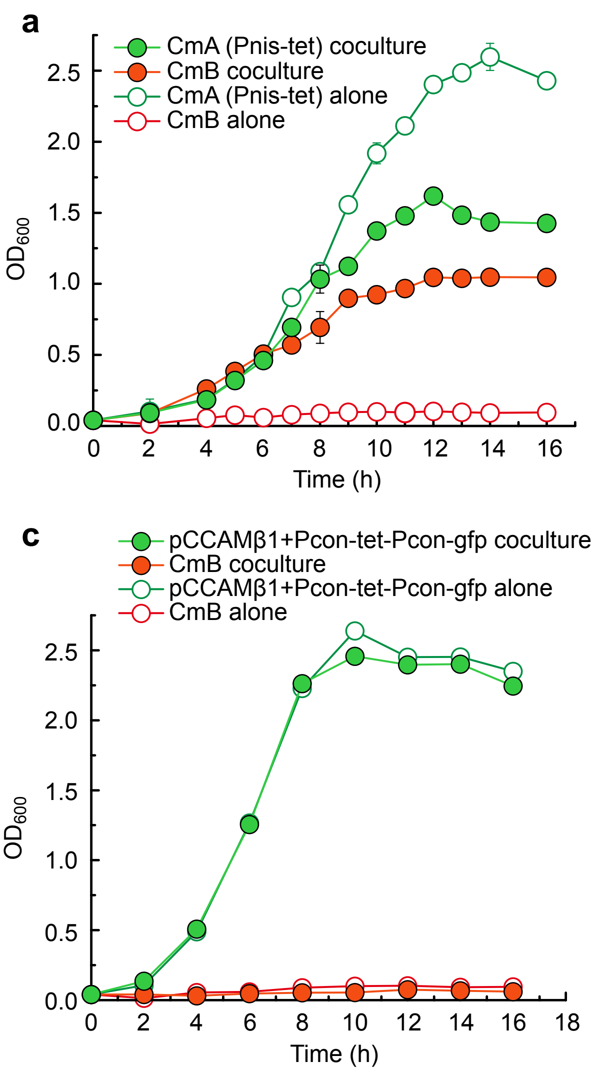

Supplementary Figure 2: Control experiments for commensalism.
(**a**) Growth curve of another case of commensalism. CmA was replaced by a strain with nisin-inducible tet^R gene. Self-inducible CmA (Pnis-tet) leads to a slower growth of itself and correspondingly a higher ratio of CmB compared to constitutively produced tet^R. Closed circles: coculture. Open circles: growth of a single strain. (**b**) Growth curve of a single strain of commensalism in GM17 media with erythromycin and tetracycline (Tet). Close circles represent induced with 25 ng/ml of nisin at the beginning and open circles represent uninduced. (**c**) CmA was replaced with a control strain unable to benefit CmB. Closed circles: growth in mixed population. Open circles: growth of a single strain. Data are presented as mean (s.d.), n=3.

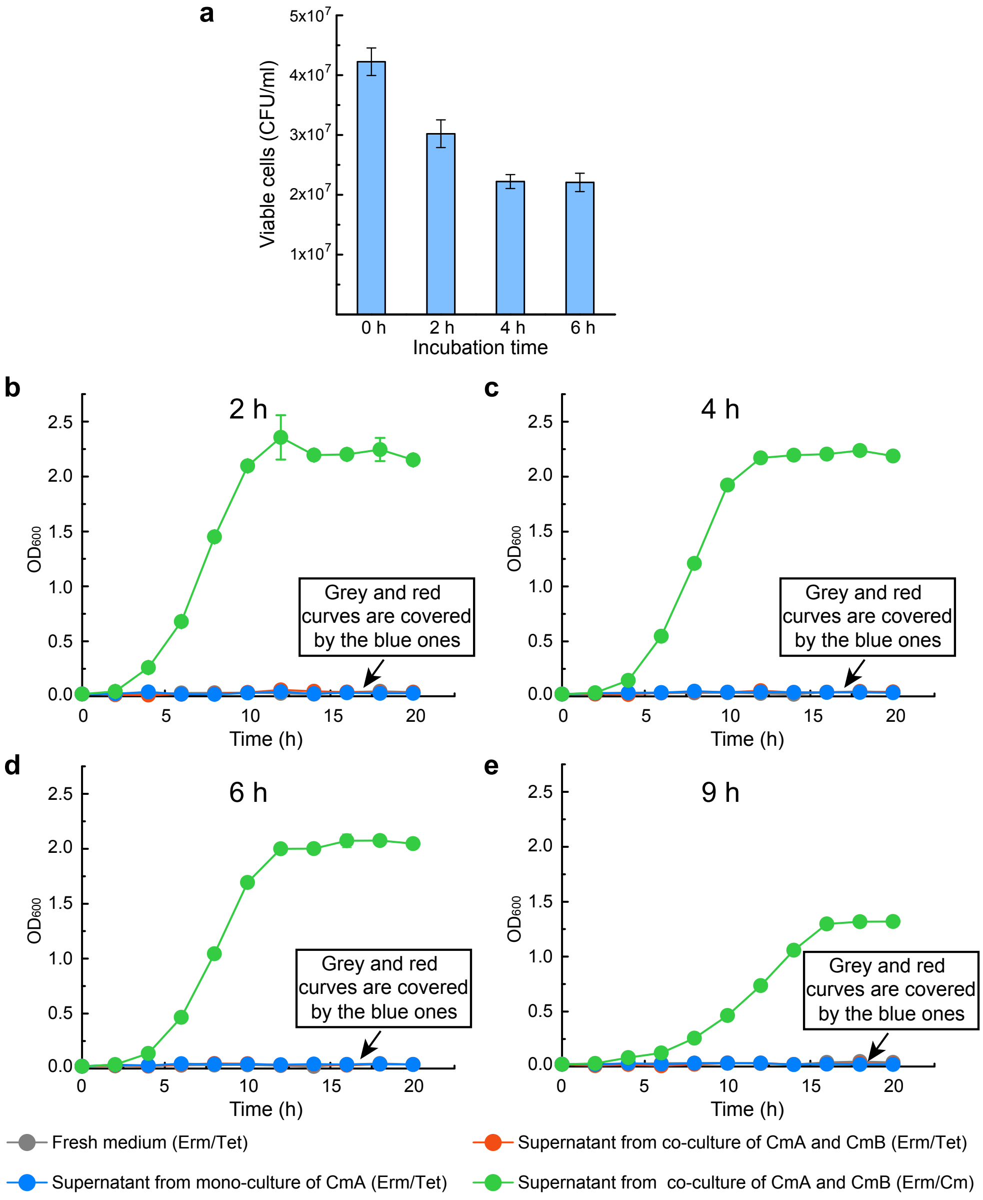

Supplementary Figure 3: Impact of tetracycline on the commensalism system.
(**a**) Survival of CmB after incubation with tetracycline. CmB was inoculated in GM17 medium supplemented with erythromycin (Erm, 5 μg/ml) and tetracycline (Tet, 10 μg/ml) to a final OD_{600} of 0.2. The culture was incubated at 30°C and samples were taken every two hours and viable cells were counted by plate pouring and colony counting. (**b-e**) Testing the remaining tetracycline in the coculture of CmA and CmB at the following time points: 2 hours (**b**), 4 hours (**c**), 6 hours (**d**), and 9 hours (**e**). Supernatants from coculture of CmA and CmB (grown in GM17/Erm/Tet), coculture of CmA and CmB (grown in GM17/Erm/Cm), and monoculture of CmA (grown in GM17/Erm/Tet) were collected at different time points and sterilized by filter and mixed with equal volume of GM17/Erm medium. The mixed media containing the supernatants were inoculated with CpA (resistant to Erm and nisin in the supernatant, but sensitive to Tet) cells at a final OD600 of 0.01. The growth curves were measured to test whether the concentration of remaining Tet in the supernatant was high enough to inhibit the growth of Tet-sensitive cells. As a positive control, fresh GM17/Erm/Tet was also mixed with equal volume of GM17/Erm to perform this experiment.

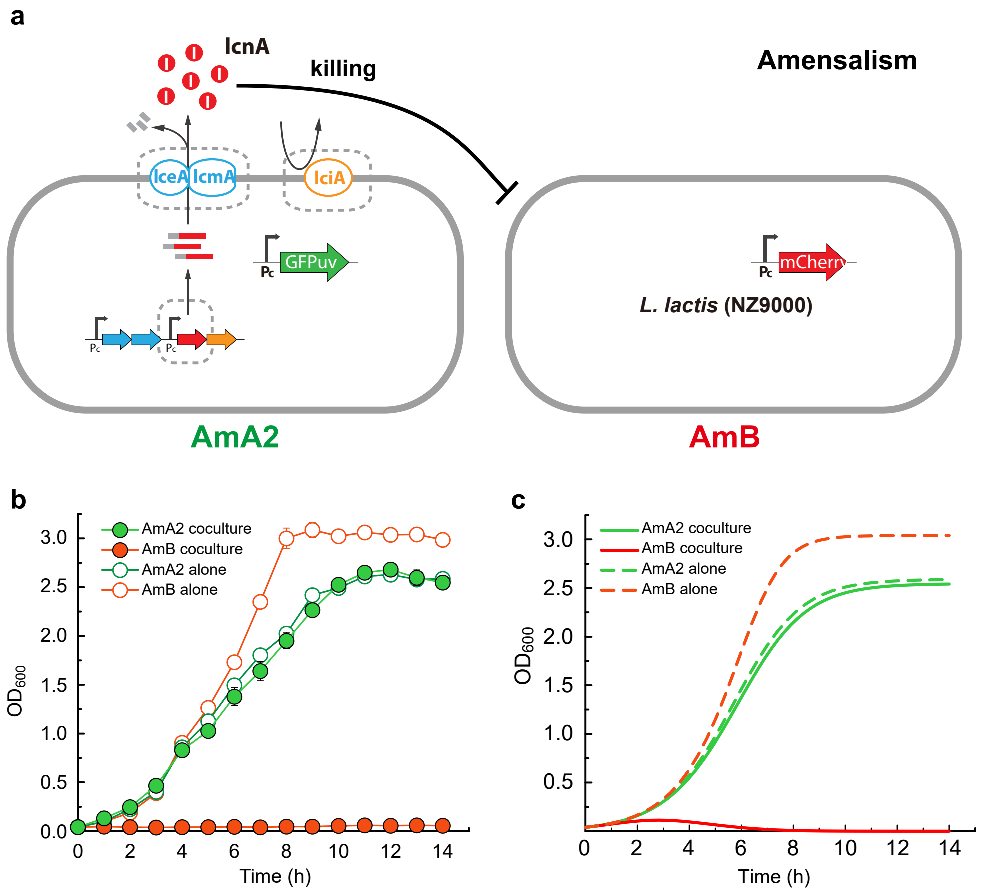

Supplementary Figure 4: Alternative design of amensalism.
(**a**) Ecosystem design. Strain A (AmA2) is a lactococcin A producer that inhibits the growth of *L. lactis* NZ9000 (AmB). (**b**) Experimental validation of the ecosystem. Data are presented as mean (s.d.), n=3. (**c**), Simulation results. Closed circles: coculture. Open circles: growth of a single strain.

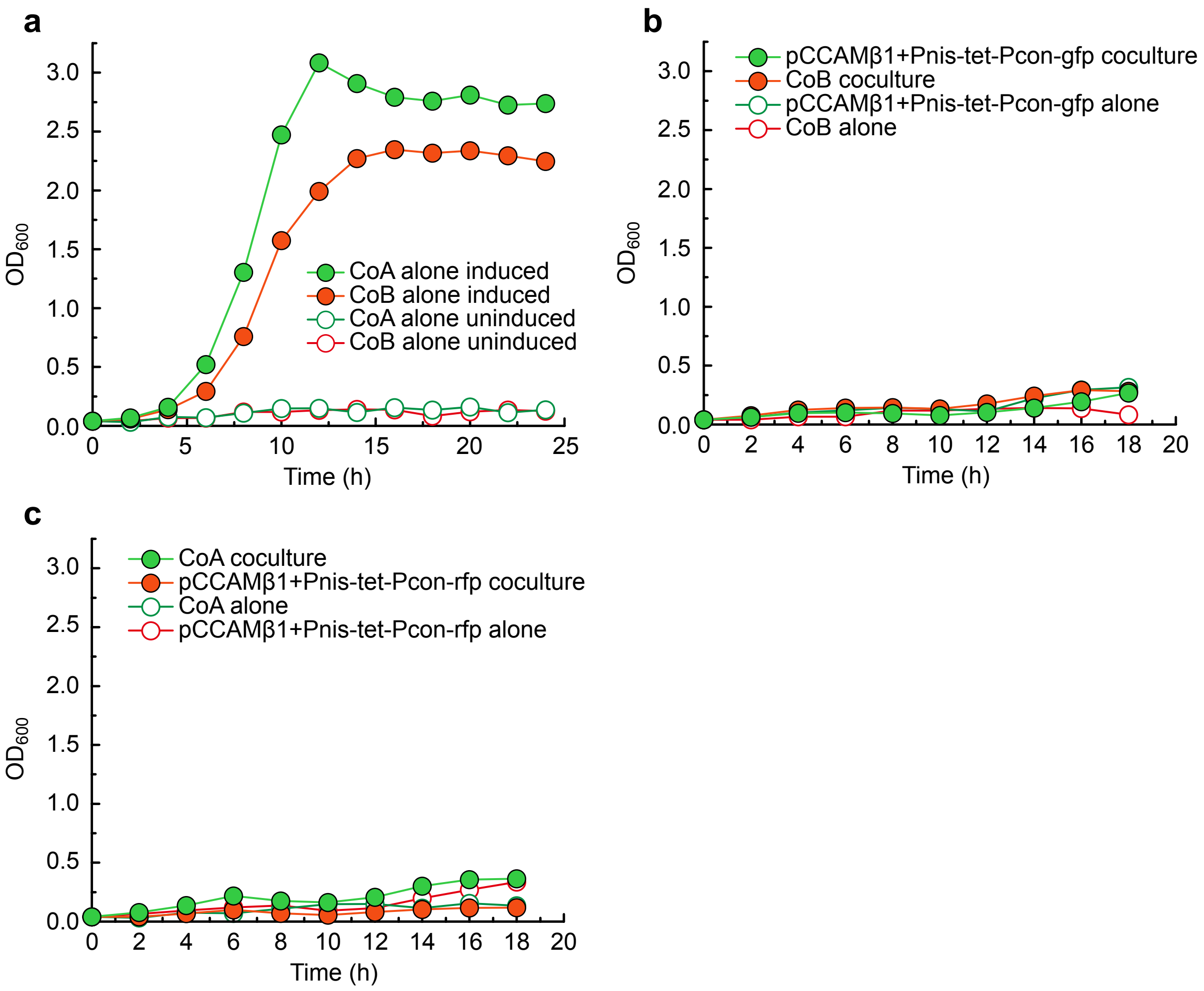

Supplementary Figure 5: Control experiments for cooperation.
(**a**) Growth curve of a single strain of cooperation in GM17 media with erythromycin and tetracycline. Close circles represent induced with 25 ng/ml of nisin at the beginning and open circles represent uninduced. (**b**) CoA was replaced with a control strain that was unable to produce nisin precursor. Neither control nor CoB was able to grow because they could not cooperate to produce nisin. Closed circles: coculture. Open circles: growth of a single strain. (**c**) CoB was replaced with a control strain that was unable to produce NisP. Lack of cooperation, CoA and control did not grow. Closed circles: coculture. Open circles: growth of a single strain. Data is presented as mean (s.d.), n=3.

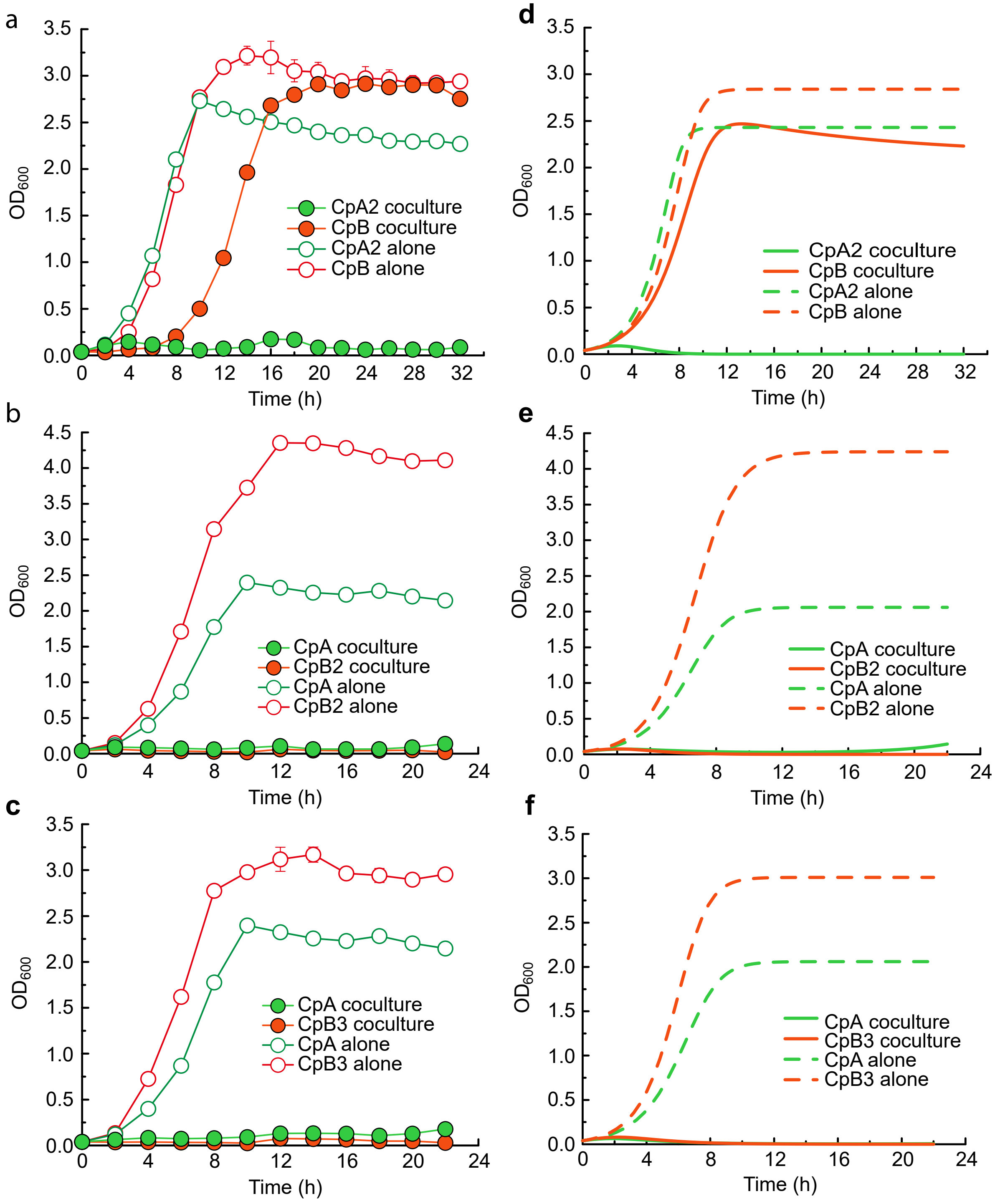

Supplementary Figure 6: Other cases of competition.
(**a**) CpB wins in the contest if CpA is a low nisin producer. (**b-c**) Close contest between CpA and CpB. (**d-f**) Simulation results of a-c. Closed circles: coculture. Open circles: growth of a single strain. Data is presented as mean (s.d.), n=3.

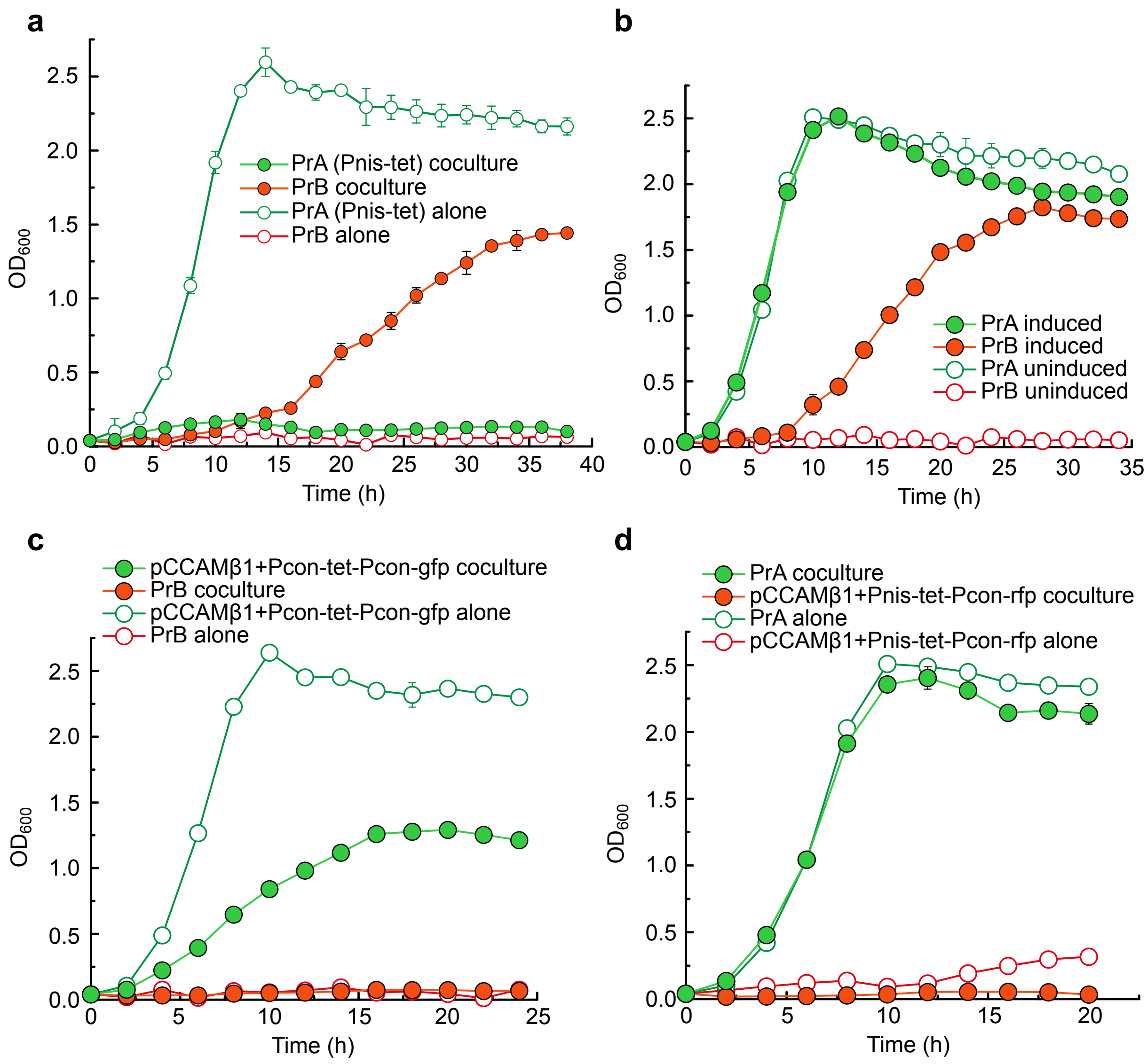

Supplementary Figure 7: Control experiments for predation.
(**a**) Growth curve of another case of predation. PrA (Pnis-tet) needs self-induction to grow in GM17 media with erythromycin and tetracycline, which leads to a slower growth of PrA(Pnis-tet). Closed circles: coculture. Open circles: growth of a single strain. (**b**) Growth curve of a single strain of predation in GM17 media with erythromycin and tetracycline. Close circles represent induced with 25 ng/ml of nisin at the beginning and open circles represent uninduced. (**c**) A control experiment that PrA (prey) was unable to produce nisin to benefit PrB. (**d**) A control experiment that PrB (predator) was unable to kill PrA. The control strain grew worse in coculture than in mono culture because nisin produced by PrA inhibited the control strain. Closed circles: growth in coculture. Open circles: growth in mono culture. Data is presented as mean (s.d.), n=3.

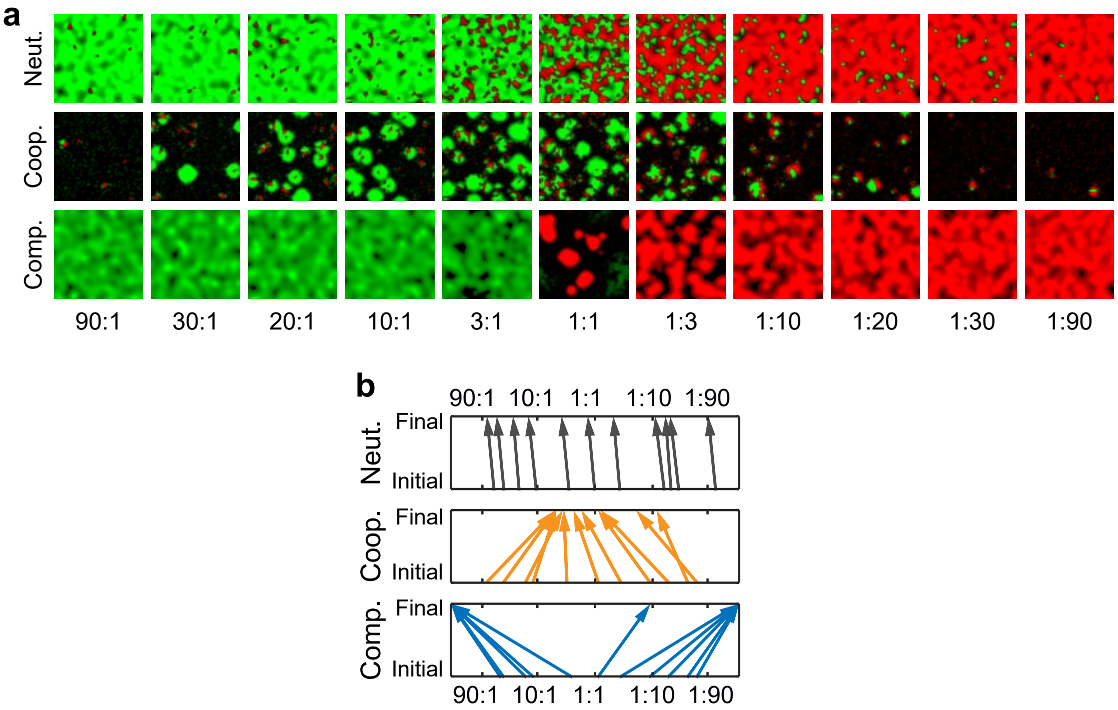

Supplementary Figure 8: Simulated Spatial dynamics of three symmetrical communities.

(**a**) Simulated spatial patterns for the consortia of neutralism, cooperation and competition. Different ratios of initial populations were used. (**b**) Evolution of the population ratios during the simulations of pattern development in panel **a**.

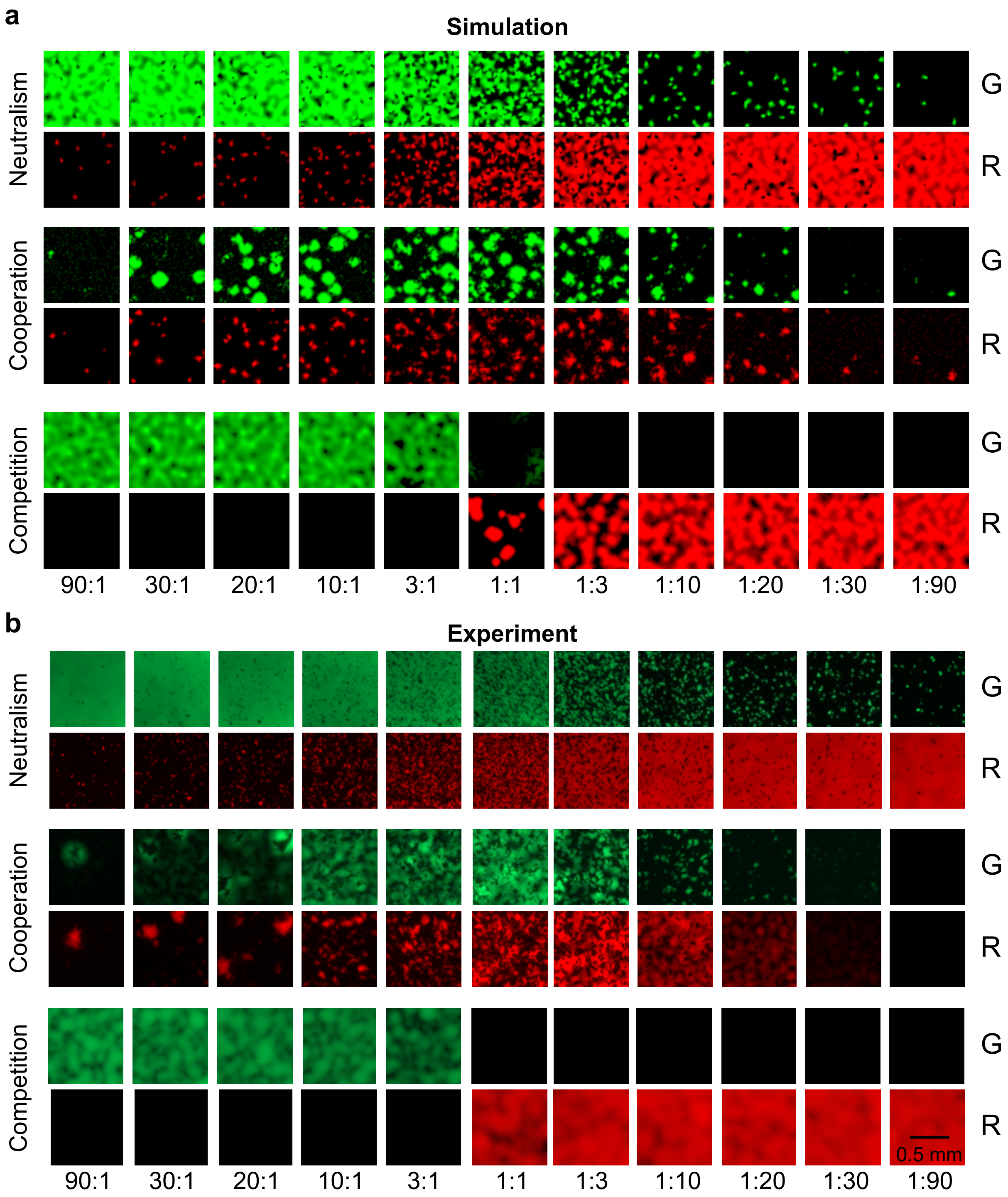

Supplementary Figure 9: Separate green and red channels of well mixed spatial patterns of neutralism, cooperation and competition with different initial ratios.
(**a**) Green and red channels shown separately from the simulation results in Supplementary Fig. 8a. (**b**) Green and red shown channels separately from the experimental results in Fig. 6a.

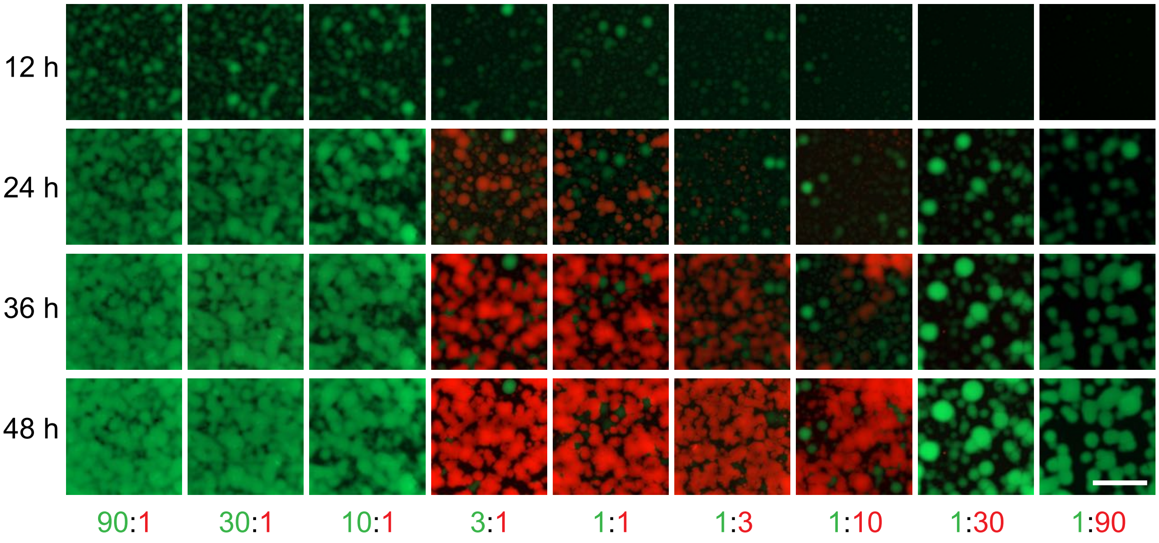

Supplementary Figure 10: Spatiotemporal patterns produced by the predation consortium on solid agar.
Overnight cultures of PrA and PrB were adjusted to the cell density of $OD_{600} = 0.2$ and mixed at ratios from 90:1 to 1:90. One microliter of cell mix was spotted onto GM17/Erm/Tet agar (2% agar) and incubated at 30°C. Images were captured every 12 hours. Each experiment was repeated at least three times. Scale bar: 1 mm. For the initial ratios from 3:1 to 1:10, PrA (green) grew first in the first 12 hours, which in turn induced PrB (red) to grow. Subsequently, induction of PrB growth released toxins that killed PrA. For initial ratios from 90:1 to 10:1, the high initial abundance and autonomous growth ability of PrA (green) enabled it to establish absolute dominance by occupying space and consuming nutrient needed for PrB (red) to grow. Therefore, no PrB was observed in this scenario. For initial ratios from 1:30 to 1:90, the extremely low concentration of PrA (green) led to failure in producing sufficient nisin which was mandatory for PrB (red) to survive. As a result, PrB went extinct and eventually only PrA (green) grew in the whole space.

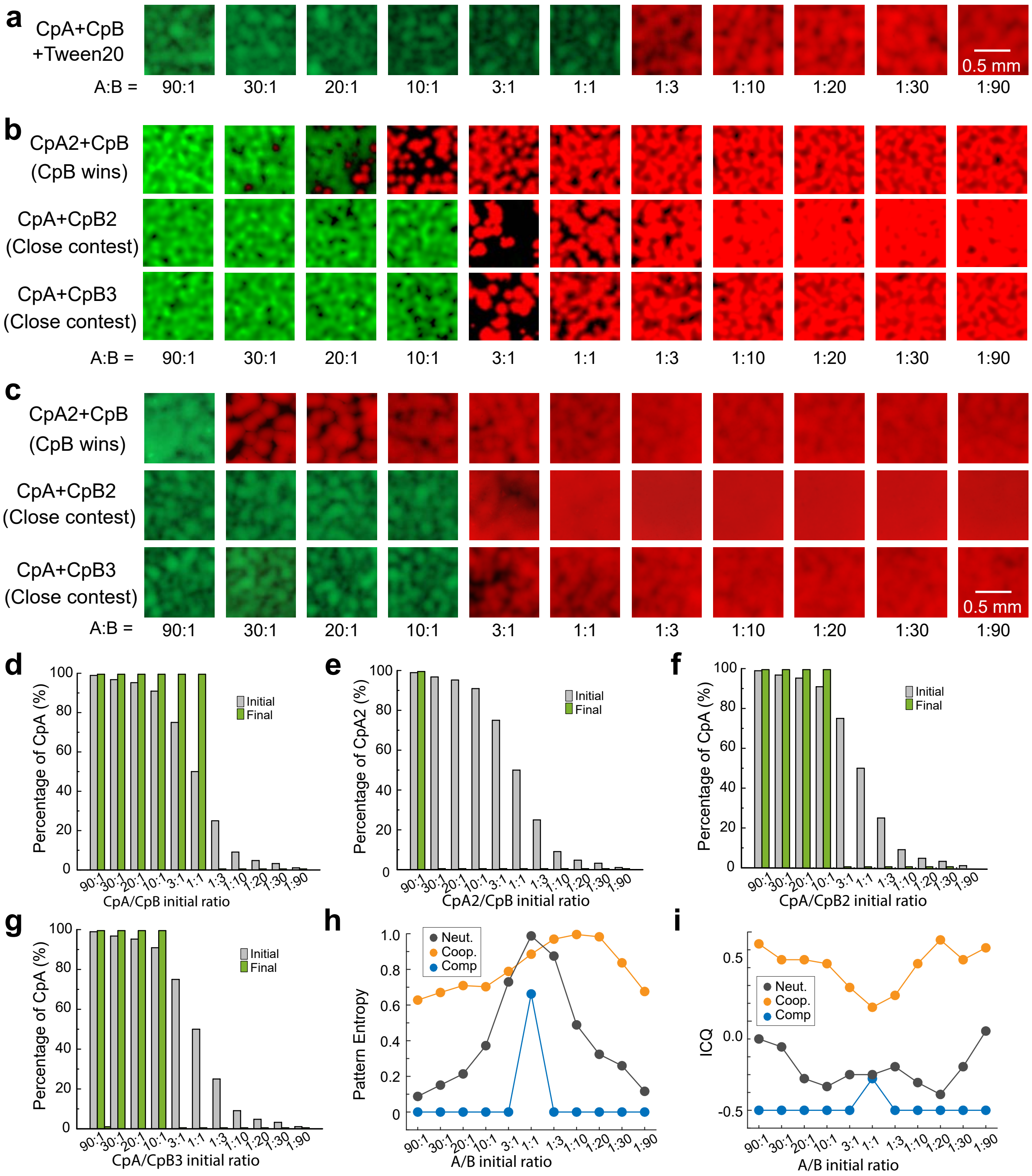

a
CpA+CpB
+Tween20
A:B =
90:1
30:1
20:1
10:1
3:1
1:1
1:3
1:10
1:20
1:30
1:90
0.5 mm
b
CpA2+CpB
(CpB wins)
CpA+CpB2
(Close contest)
CpA+CpB3
(Close contest)
c
CpA2+CpB
(CpB wins)
CpA+CpB2
(Close contest)
CpA+CpB3
(Close contest)
d
Percentage of CpA (%)
CpA/CpB initial ratio
Initial
Final
e
Percentage of CpA2 (%)
CpA2/CpB initial ratio
f
Percentage of CpA (%)
CpA/CpB2 initial ratio
g
Percentage of CpA (%)
CpA/CpB3 initial ratio
h
Pattern Entropy
A/B initial ratio
Neut.
Coop.
Comp
i
ICQ
A/B initial ratio

Supplementary Figure 11: Additional experiments and analyses of spatial ecosystem patterns.
(**a**) Nisin and lcnA have distinct diffusion constants due to the more hydrophobic nature of the former. In liquid culture experiment, the difference of the diffusion constants does not matter as the molecules are always well-mixed. However, in the settings of agar plates, the nisin producer (CpA) is much less effective in killing the lcnA producer (CpB), which resulted in different outcomes of 1:1 ratio competition in culture and in space (CpA won in culture but lost in agar, Figs. 3d and 6a). (**b-c**) Other cases of spatial patterns from competition. (**b**) Simulations of competition for the cases of strain B wins and two close contests. (**c**) Experimental results of the cases of B wins and two close contests on solid agar. (**d**) Initial and final ratios of CpA in the case of "A wins" with Tween 20. The analysis corresponds to the experimental result in panel A. Data is presented as mean (s.d.), n=3. (**e**) Initial and final ratios of CpA2 in the case of B wins, corresponding to the experimental result in panel c (top row). Data is presented as mean (s.d.), n=3. (**f**) Initial and final ratios of CpA in a case of close contest (CpA+CpB2), corresponding to the experimental result in panel c (middle row). Data is presented as mean (s.d.), n=3. (**g**) Initial and final ratios of CpA in another case of close contest (CpA+CpB3), corresponding to the experimental result in panel c (bottom row). Data is presented as mean (s.d.), n=3. (**h**) Entropy for the simulated patterns of the ecosystems. Competition has no entropy because one strain wipes out the other except the 1:1 case. Neutralism retains the entropy from the initial conditions. Cooperation increases entropy. (**i**) Intensity Correlation Quotient for the simulated patterns of the ecosystems. Cooperation is more colocalized than neutralism which is more colocalized that competition. The experiment agrees visually with the simulation.

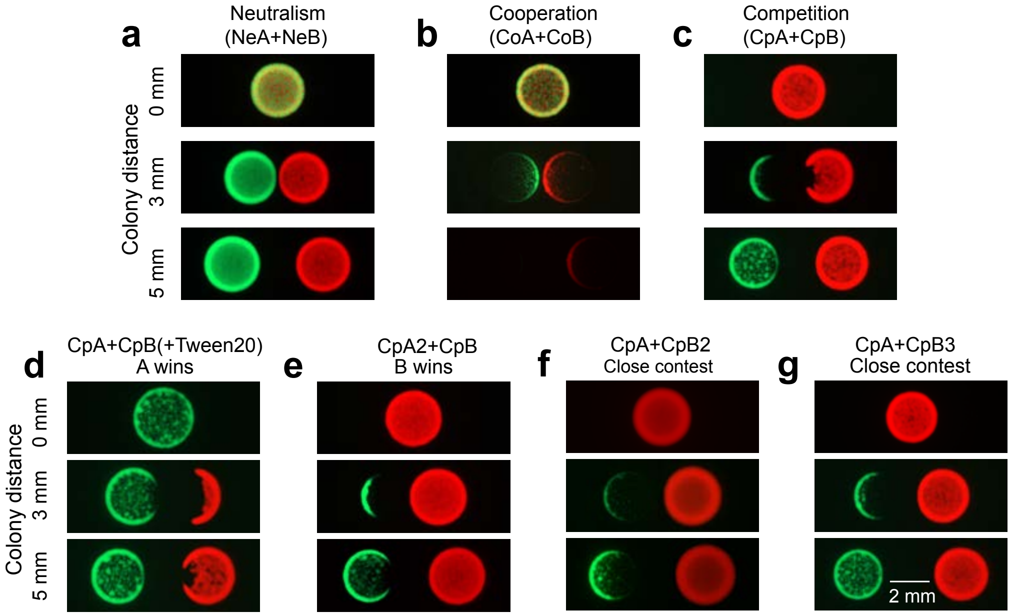

Supplementary Figure 12: Spatial patterns of communities in structured settings. The three consortia (neutralism, cooperation and competition) produced distinct spatial structures when the droplets of individual strains were plated with altered distance (0 mm, 3 mm and 5 mm). (**a**), NeA and NeB always coexisted although increasing their spacing separated them. (**b**), CoA and CoB coexisted but their pattern developments decayed with the spacing. In the case of 5 mm spacing, the nisin precursors produced by CoA can diffuse to the sites of CoB and then be modified by CoB to become active nisin. These active nisin can be directly utilized by CoB due to close proximity; in contrast, CoA cannot utilize the active nisin unless it diffuses back to the sites where CoA locates. Therefore, only red cells (CoB) were observed. (**c**), CpA and CpB were mutually excluded but, as their spacing increases, coexistence increased. CpB (red strain) won the CpA-CpB contest in structured colonies, contradicting to the culture experiment of the same strains where CpA won. This is due to the difference of the diffusion rates of nisin and lcnA in agar plates. (**d**) The case of "A wins" (CpA+CpB) with Tween 20, which facilitates nisin diffusion. (**e**) The case of "B wins" (CpA2+CpB). (**f**) A case of close contest (CpA+CpB2). (**g**) Another case of close contest (CpA+CpB3). Each experiment was repeated at least three times. Representative pictures from experiments are shown.

Supplementary Note 1: Liquid Culture Ecosystem Modeling

General Modeling Framework

Experimentally we constructed the six ecological systems by using existing cell systems in multiple ways to produce multiple interactions. We therefore simulated the ecosystems modularly; if the same cell system was used in multiple experiments, the same equation module was used in multiple simulations. To capture the essential features of our ecosystems we developed an ordinary differential equation (ODE) model consisting of two bacterial strains, two signaling molecules, and a common nutrient. Each strain grows by consuming the common nutrient and interacts with the other strain through signaling molecules. The general form of the equations is:

$$\begin{aligned}
\frac{dN_1}{dt} &= g_1(F)N_1 - I_1(S_1,S_2)N_1 \\
\frac{dN_2}{dt} &= g_2(F)N_2 - I_2(S_1,S_2)N_2 \\
\frac{dS_1}{dt} &= P_1(S_1,S_2,N_1,N_2,F) - k_1S_1 \\
\frac{dS_2}{dt} &= P_2(S_1,S_2,N_1,N_2,F) - k_2S_2 \\
\frac{dF}{dt} &= -\frac{1}{\gamma_1}G_1(F,S_1,S_2)N_1 - \frac{1}{\gamma_2}G_2(F,S_1,S_2)N_2
\end{aligned} \tag{S1}$$

Two bacterial strains N_1 and N_2 consume common nutrient F. Nutrient consumption is modeled with growth functions g_1 and g_2. Two yield parameters γ_1 and γ_2 measure the number of bacteria produced from the nutrient supply. Two signaling molecules S_1 and S_2 are produced by bacteria. Signaling molecule production (P_1, P_2) depends on the concentration of signaling molecules, the populations of bacterial cells and the concentration of nutrient. Decay of signaling molecules is approximated by first order kinetics. Once produced, the signaling molecules can either help or harm bacterial growth. Intermediate precursor species may be introduced when the synthesis of a signaling molecule requires multiple strains. The interaction between the signaling molecules and the bacteria is described by interaction functions I_1 and I_2. Nutrient reduction (G_1, G_2) describes net nutrient loss due to consumption for cell growth and recycling from cell death via cellular interactions. All six ecosystems feature different interactions.

The general framework Eq. $S1$ describes all six ecosystems. For reference, a top level presentation of all six ecosystems follows.

Commensalism

$$\begin{aligned}
\frac{dN_1}{dt} &= g_1(F)N_1 \\
\frac{dN_2}{dt} &= g_2(F)N_2 - I_t(S_n)N_2 \\
\frac{dS_n}{dt} &= P_s(S_n,F)N_1 - k_nS_n \\
\frac{dF}{dt} &= -\frac{1}{\gamma_1}G_1N_1 - \frac{1}{\gamma_2}G_2N_2
\end{aligned} \tag{S2}$$

Amensalism

$$\begin{aligned}
\frac{dN_1}{dt} &= g_1(F)N_1 \\
\frac{dN_2}{dt} &= g_2(F)N_2 - I_n(S_n)N_2 \\
\frac{dS_n}{dt} &= P_s(S_n, F)N_1 - k_n S_n \\
\frac{dF}{dt} &= -\frac{1}{\gamma_1}G_1 N_1 - \frac{1}{\gamma_2}G_2 N_2
\end{aligned} \tag{S3}$$

Neutralism

$$\begin{aligned}
\frac{dN_1}{dt} &= g_1(F)N_1 \\
\frac{dN_2}{dt} &= g_2(F)N_2 \\
\frac{dF}{dt} &= -\frac{1}{\gamma_1}G_1 N_1 - \frac{1}{\gamma_2}G_2 N_2
\end{aligned} \tag{S4}$$

Cooperation

$$\begin{aligned}
\frac{dN_1}{dt} &= g_1(F)N_1 - I_{t,1}(S_n)N_1 \\
\frac{dN_2}{dt} &= g_2(F)N_2 - I_{t,2}(S_n)N_2 \\
\frac{dS_p}{dt} &= P_s(S_n, F)N_1 - k_m S_p N_2 - k_p S_p \\
\frac{dS_n}{dt} &= k_m S_p N_2 - k_n S_n \\
\frac{dF}{dt} &= -\frac{1}{\gamma_1}G_1 N_1 - \frac{1}{\gamma_2}G_2 N_2
\end{aligned} \tag{S5}$$

Competition

$$\begin{aligned}
\frac{dN_1}{dt} &= g_1(F)N_1 - I_l(S_l)N_1 \\
\frac{dN_2}{dt} &= g_2(F)N_2 - I_n(S_n)N_2 \\
\frac{dS_n}{dt} &= P_s(S_n, F)N_1 - k_n S_n \\
\frac{dS_l}{dt} &= r_l N_2 F - k_l S_l \\
\frac{dF}{dt} &= -\frac{1}{\gamma_1}G_1 N_1 - \frac{1}{\gamma_2}G_2 N_2
\end{aligned} \tag{S6}$$

Predation

$$\begin{aligned}
\frac{dN_1}{dt} &= g_1(F)N_1 - I_l(S_l)N_1 \\
\frac{dN_2}{dt} &= g_2(F)N_2 - I_t(S_n)N_2 \\
\frac{dS_n}{dt} &= P_s(S_n, F)N_1 - k_n S_n \\
\frac{dS_l}{dt} &= r_l N_2 F - k_l S_l \\
\frac{dF}{dt} &= -\frac{1}{\gamma_1}G_1 N_1 - \frac{1}{\gamma_2}G_2 N_2
\end{aligned} \tag{S7}$$

Modules used in the six models:

Growth

$$\begin{aligned}
g_1(F) &= \mu_1 \frac{F}{K_1 + F} \\
g_2(F) &= \mu_2 \frac{F}{K_2 + F}
\end{aligned} \tag{S8}$$

Interactions

$$\begin{aligned}
I_t(S_n) &= d_t \frac{1}{1 + k_s S_n} \\
I_n(S_n) &= d_{S_n} \frac{S_n}{K_{S_n} + S_n} \\
I_l(S_l) &= d_{S_l} \frac{S_l}{K_{S_l} + S_l}
\end{aligned} \tag{S9}$$

Signaling Molecule Production

$$P_s(S_n, F) = \alpha \frac{S_n + r_b}{K_p + S_n} F \tag{S10}$$

Additionally, nutrient reduction terms (G_1, G_2) in the above systems describe net nutrient loss due to nutrient consumption for cell growth and recycling from cell death of corresponding strains. Their specific expressions are $g_1(F)$ and $g_2(F) - \varepsilon_t I_t(S_n)$ for commensalism, $g_1(F)$ and $g_2(F) - \varepsilon_n I_n(S_n)$ for amensalism, $g_1(F)$ and $g_2(F)$ for neutralism, $g_1(F) - \varepsilon_t I_{t,1}(S_n)$ and $g_2(F) - \varepsilon_t I_{t,2}(S_n)$ for cooperation, $g_1(F) - \varepsilon_l I_l(S_l)$ and $g_2(F) - \varepsilon_n I_n(S_n)$ for competition, $g_1(F) - \varepsilon_l I_l(S_l)$ and $g_2(F) - \varepsilon_t I_t(S_n)$ for predation respectively, where ε's are recycling coefficients.

Commensalism

Commensalism happens when one strain produces a signal which improves the growth of another strain. To produce commensalism we made use of an antibiotic and a signal which induces antibiotic resistance. We grew two nisin-immune types of bacteria in an environment supplemented with tetracycline (Tet). In our system the green strain produces nisin and is naturally Tet-immune, the red strain is killed by Tet unless the strain senses nisin. Once sensed, nisin causes the red bacteria to produce Tet^R, a protein which pumps Tet out of the cell (*5*). Tet inhibits the ribosomes

of bacteria, slowing down growth or, at high concentrations, killing bacteria. In our experiment Tet concentrations do not quickly kill bacteria. Similar to how nisin and lactococcin A (lcnA) kill bacteria, we model tetracycline killing bacteria with a hill function.

$$\frac{dN}{dt} = -d_t \frac{T_i}{K_t + T_i} N \tag{S11}$$

where d_t is the maximal death rate. K_t is the Michaelis constant. Lastly T_i represents the concentration of Tet inside a bacterial cell.

Tet is assumed to diffuse into cells according to first order kinetics. Tet inside the cell is pumped out by Tet^R.

Our model assumes the rate of pumping Tet out of the cell is proportional to the concentration of Tet^R which is, in turn, proportional to nisin availability (S_n). Thus,

$$\frac{dT_i}{dt} = -k_s S_n T_i + k(T - T_i) \tag{S12}$$

where k_s, k are rate constants and T is the extracellular concentration of Tet. Using the steady state assumption and combining constants we arrive at the equation

$$T_i = \frac{T}{k_s S_n + 1} \tag{S13}$$

where k is absorbed into k_s. Combining Eqs. $S11$ and $S13$ and absorbing K_t into T result in a commensalistic social interaction $I_t(S_n)$.

$$\frac{dN}{dt} = -d_t \frac{T}{1 + T + k_s S_n} N = -I_t(S_n) N \tag{S14}$$

The equation has more parameters than needed. We absorb T and $1+T$ into d_t and k_s, and rewrite the equation as

$$\frac{dN}{dt} = -d_t \frac{1}{1 + k_s S_n} N = -I_t(S_n) N \tag{S15}$$

If we denote the green strain as strain 1 and the red strain as strain 2 then commensalism arises by combining strain growth (described in neutralism), nisin production (described in amensalism), and nisin-induced Tet immunity (Eqs. $S32$, $S18$, $S15$).

$$\begin{aligned} \frac{dN_1}{dt} &= g_1(F) N_1 \\ \frac{dN_2}{dt} &= g_2(F) N_2 - I_t(S_n) N_2 \\ \frac{dS_n}{dt} &= P_s(S_n, F) N_1 - k_n S_n \\ \frac{dF}{dt} &= -\frac{1}{\gamma_1} G_1 N_1 - \frac{1}{\gamma_2} G_2 N_2 \end{aligned} \tag{S16}$$

Amensalism Nisin

Amensalism occurs when one strain hurts the growth of another strain. Modeling amensalism required a system where one bacterial strain harms the other bacterial strain through a signaling molecule. Our experimental design was that green bacteria produce nisin which harms red bacteria. We refer to nisin as a signaling molecule because nisin is produced by one strain and induces a signal, in this case death, in another strain.

Nisin production was assumed to be produced both through quorum sensing and at a basal rate. (*4*) (*6*) It is a function of nisin concentration, nutrient concentration, and green bacteria concentration.

$$\frac{dS_n}{dt} = \alpha \frac{S_n + r_b}{K_p + S_n} NF - k_n S_n \tag{S17}$$

S_n is the signaling molecule nisin, α describes the maximum rate of production, K_p is the Michaelis constant, r_b is the basal production rate, and k_n describes decay of nisin with time.

To simplify and show the modularity, Eq. $S17$ will be written as:

$$\frac{dS_n}{dt} = P_s(S_n, F)N - k_n S_n \tag{S18}$$

with P_s standing for production of nisin.

Nisin works on cells by opening small holes in cell walls of bacteria which allows nutrients, proteins, and other intracellular chemicals to seep out of the cells. (*7*) (*8*) (*9*) At low concentrations nisin acts as a bacteriocin, slowing or stopping cell growth. At higher concentrations nisin kills cells. A hill function is used to describe cell loss. (*10*) (*11*)

$$\frac{dN}{dt} = -d_{S_n} \frac{S_n}{K_{S_n} + S_n} N \tag{S19}$$

At low concentrations of nisin S_n the bacterial growth is suppressed. At high nisin concentrations the death rate is larger than the growth term and bacteria start dying. To simplify the notation Eq. $S19$ is simplified to:

$$\frac{dN}{dt} = -I_n(S_n)N \tag{S20}$$

with I_n describes the interaction due to nisin.

Denoting the green strain as strain 1 and the red strain as strain 2 and combining the equations for two strain growth (described in neutralism), nisin production, and antibiotic action (Eqs. $S32$, $S18$, $S20$) we arrive at.

$$\begin{aligned}
\frac{dN_1}{dt} &= g_1(F)N_1 \\
\frac{dN_2}{dt} &= g_2(F)N_2 - I_n(S_n)N_2 \\
\frac{dS_n}{dt} &= P_s(S_n, F)N_1 - k_n S_n \\
\frac{dF}{dt} &= -\frac{1}{\gamma_1}G_1 N_1 - \frac{1}{\gamma_2}G_2 N_2
\end{aligned} \tag{S21}$$

Amensalism LcnA

A second amensalism system was produced experimentally with lcnA. In our second setup green bacteria produce lcnA which harms red bacteria.

LcnA is constitutively produced in bacteria and decays according to first order kinetics (*12*). Therefore lcnA concentration obeys:

$$\frac{dS_l}{dt} = r_l NF - k_l S_l \tag{S22}$$

with S_l being the signaling molecule lcnA. Parameter r_l models constitutive production of S_l and parameter k_l describes lcnA decay.

We used the same Hill function form to model the antibiotic action of lcnA as we used for nisin. A lcnA susceptible cell is then killed according to:

$$\frac{dN}{dt} = -d_{S_l}\frac{S_l}{K_{S_l} + S_l}N \tag{S23}$$

To simplify the notation, equation Eq. $S23$ will be written as:

$$\frac{dN}{dt} = -I_l(S_l)N \tag{S24}$$

Denoting the green strain as strain 1 and the red strain as strain 2 and combining the equations for neutralism (Eq. $S32$) with the modules describing lcnA we arrive at:

$$\begin{aligned}
\frac{dN_1}{dt} &= g_1(F)N_1 \\
\frac{dN_2}{dt} &= g_2(F)N_2 - I_l(S_l)N_2 \\
\frac{dS_l}{dt} &= r_l N_1 F - k_l S_l \\
\frac{dF}{dt} &= -\frac{1}{\gamma_1}G_1N_1 - \frac{1}{\gamma_2}G_2N_2
\end{aligned} \tag{S25}$$

This strain is omitted from the outline of the six ecosystems because its behavior is not qualitatively different from amensalism with nisin.

Neutralism

Bacterial population growth was modeled by a Monod function (*13*); growth of a single strain has the form

$$\frac{dN}{dt} = \mu_{max}N\frac{F}{K + F} \tag{S26}$$

Parameters μ_{max} and K determine the maximum growth rate and the half concentration value.

Nutrient concentration starts at a dimensionless value of 1. Following (*13*), consumption of nutrients is proportional to growth. A yield parameter γ linearly relates growth and consumption:

$$\gamma\frac{dF}{dt} = -\frac{dN}{dt} \tag{S27}$$

Combining growth and nutrient consumption produces a model of single strain growth.

$$\frac{dN}{dt} = \mu_{max}N\frac{F}{K + F} \tag{S28}$$

$$\gamma\frac{dF}{dt} = -\frac{dN}{dt} \tag{S29}$$

To simplify the notation we denote Monod consumption as $g(F)$. After simplifying the equations Eq. $S28$ and Eq. $S29$ become:

$$\frac{dN}{dt} = g(F)N \tag{S30}$$

$$\frac{dF}{dt} = -\frac{1}{\gamma}g(F)N \tag{S31}$$

Neutralism occurs in our design when two bacteria indirectly compete for nutrients but there are no direct interactions. In modeling neutralism, growth is assumed to have the same form as for a single strain, but with a common nutrient. Therefore we need to take Eqs. $S30$ and $S31$ and apply them to two different strains with a common nutrient.

The resulting equations are

$$\begin{aligned} \frac{dN_1}{dt} &= g_1(F)N_1 \\ \frac{dN_2}{dt} &= g_2(F)N_2 \\ \frac{dF}{dt} &= -\frac{1}{\gamma_1}G_1N_1 - \frac{1}{\gamma_2}G_2N_2 \end{aligned} \tag{S32}$$

Functions $g_1(F)$ and $g_2(F)$ are Monod functions of a common nutrient. Growth and nutrient consumption follows the same format for all ecosystems. Nutrient reductions (G_1, G_2) describe net nutrient loss due to consumption for cell growth and recycling from cell death, which are subject to growth and cellular interactions and, for neutralism, equal the growth rates ($g_1(F)$, $g_2(F)$).

Cooperation

Cooperation arises when two strains benefit each other. Our cooperative system is obligatory; each strain can barely grow without the other strain. To develop cooperative strains, we utilized many modules from the commensalism strains, however now two strains (green and red) are tailored to produce nisin. Additionally both strains are killed by Tet unless they work together to produce nisin.

In all the previous experiments nisin production was a multistep process within a single cell (*14*). Prenisin was produced inside of cells, and then excreted. Once outside the cell, prenisin is modified by an enzyme nisP which removes excess amino acids and modifies existing amino acids to produce mature nisin. For commensalism the green strain both produced nisin and modified it. To produce a mutualistic interaction we moved the nisP gene to the red strain. Therefore the green strain produces prenisin and the red strain turns prenisin into mature nisin.

Similar to commensalism the environment contains Tet, which harms both strains. Any nisin causes both the red and green strains to produce Tet^R. Once produced, Tet^R will pump Tet out of cells allowing the bacteria to grow.

Production of prenisin is modeled the same way mature nisin was produced in the other simulations.

$$\frac{dS_p}{dt} = P_s(S_n, F)N \tag{S33}$$

where S_p represents prenisin. Assuming first order reaction rate, prenisin is modified into nisin according to:

$$\frac{dS_n}{dt} = k_mS_pN \tag{S34}$$

where k_m describes the reaction rate of nisP interacting with prenisin. Including first order degradation with a rate constant of k_p, the equation governing prenisin becomes:

$$\frac{dS_p}{dt} = P_s(S_n, F)N_1 - k_mS_pN_2 - k_pS_p \tag{S35}$$

where strain 1 produces prenisin and strain 2 modifies the prenisin into nisin. For simplicity, we assumed prenisin and nisin degrade equally ($k_p = k_n$).

Using the assumption of first order decay, we arrive at the following equation for nisin

$$\frac{dS_n}{dt} = k_m S_p N_2 - k_n S_n \tag{S36}$$

Alternatively, we can combine prenisin and nisin kinetics into a single equation as

$$\frac{dS_n}{dt} = P_m(S_n, N_1, N_2, F) - k_n S_n \tag{S37}$$

where $P_m(S_n, N_1, N_2, F) = k_m P_s(S_n, F) N_1 N_2/(k_p + k_m N_2)$. The both models are applicable but the former was adopted to be consistent with spatial modeling later.

A cooperative system is created by combining the existing equations for growth and nisin-suppressed Tet killing along with the new mutualistic equation for nisin production.

$$\begin{aligned}
\frac{dN_1}{dt} &= g_1(F)N_1 - I_{t,1}(S_n)N_1 \\
\frac{dN_2}{dt} &= g_2(F)N_2 - I_{t,2}(S_n)N_2 \\
\frac{dS_p}{dt} &= P_s(S_n, F)N_1 - k_m S_p N_2 - k_p S_p \\
\frac{dS_n}{dt} &= k_m S_p N_2 - k_n S_n \\
\frac{dF}{dt} &= -\frac{1}{\gamma_1} G_1 N_1 - \frac{1}{\gamma_2} G_2 N_2
\end{aligned} \tag{S38}$$

Competition

Competition occurs when two bacteria both produce signaling molecules which harm the other strain. Utilizing both the nisin and lcnA modules from the two types of amensalism we produced a competitive system. In our system green bacteria harm red through nisin and red bacteria harm green bacteria with lcnA.

The equations come from combining Eq. $S21$ and Eq. $S25$. Green bacteria is strain 1 and red bacteria is strain 2.

$$\begin{aligned}
\frac{dN_1}{dt} &= g_1(F)N_1 - I_l(S_l)N_1 \\
\frac{dN_2}{dt} &= g_2(F)N_2 - I_n(S_n)N_2 \\
\frac{dS_n}{dt} &= P_s(S_n, F)N_1 - k_n S_n \\
\frac{dS_l}{dt} &= r_l N_2 F - k_l S_l \\
\frac{dF}{dt} &= -\frac{1}{\gamma_1} G_1 N_1 - \frac{1}{\gamma_2} G_2 N_2
\end{aligned} \tag{S39}$$

Predation

Predation is when one strain both benefits from and harms another strain. In our system green bacteria produce nisin and are susceptible to lcnA. Red bacteria produce lcnA and become Tet-immune because of nisin.

Predation does not require introducing any new modules. Green bacteria benefiting red bacteria through nisin is already described by commensalism. Red bacteria producing lcnA to hurt green is described by amensalism lcnA. Combining commensalism and amensalism lcnA results in predation:

$$\begin{aligned}
\frac{dN_1}{dt} &= g_1(F)N_1 - I_l(S_l)N_1 \\
\frac{dN_2}{dt} &= g_2(F)N_2 - I_t(S_n)N_2 \\
\frac{dS_n}{dt} &= P_s(S_n, F)N_1 - k_n S_n \\
\frac{dS_l}{dt} &= r_l N_2 F - k_l S_l \\
\frac{dF}{dt} &= -\frac{1}{\gamma_1} G_1 N_1 - \frac{1}{\gamma_2} G_2 N_2
\end{aligned} \tag{S40}$$

Supplementary Note 2: Computational Methods

Software

All well mixed simulations were done in Matlab using the ode45 subroutine. Data fitting was done by selecting parameter sets manually to fit experimental data. Spatial simulations were simulated in C++. Matlab was used to analyze the output, analyze images, and produce figures.

Single-Strain Data Fitting and Initial Conditions

All six two-strain models have two bacterial strains, additionally the three-strain and four-strain ecosystems require the creation of additional strains. Each strain has three parameters (μ, K, and γ) which describe its growth and nutrient consumption. Growing each bacterial strain alone can determine these parameters in the cases of neutralism, amensalism, and competition.

In the cases of commensalism, cooperation, and predation tetracycline inhibits growth. To determine the growth rates in these ecosystems we grew single strain with a high concentration of nisin added.

For each single strain run we measured the population at 2 hour intervals. The data was taken and fit to equations Eq. $S28$ and Eq. $S29$. The parameters were fit for every experiment. Each experiment was repeated three times.

All single strain simulation runs start at an OD600 of 0.04. The simulation time for each simulation is the same as that of corresponding experiments. Results for neutralism, amensalism, and competition are shown in Figs. 2 and 3. Results for commensalism, cooperation, and predation are shown in Figs. 2, 3 and Supplementary Figs. 2, 5 and 7. Values are in Supplementary Tables 4, 5 and 6.

Commensalism Data Fitting and Predictions

To fit the data for commensalism first we determined the growth curves for the green and red strains. Our nisin producing green strain was grown in isolation and its parameters were fit. Additionally the red strain was grown with a large amount of added nisin. This allowed us to determine its growth curves. Also the red strain was grown without nisin to allow us to measure the parameter d_t, the death rate due to tetracycline.

The two strains grown together were then used to fit parameter k_s to determine the effectiveness of nisin on the system. Results are shown in Fig. 2 and Supplementary Fig. 2. Values are in Supplementary Table 4.

Amensalism Data Fitting and Predictions

Amensalism was tested by growing both strains separately and also growing the strains together. Monod growth and nutrient consumption were fit from the single strain experiments. Amensalism has a two parameter model of nisin killing bacteria, Eq. $S19$. These parameters were fit from the population data for the two strain runs. The fit parameters for nisin are subsequently used in the other experiments. Results are shown in Fig. 2. Values are in Supplementary Table 4.

The experimental results for AmA2 demonstrated that lcnA killed the other strain very quickly. The results were only able to provide a lower bound on the parameters describing lcnA interactions. Results are shown in Supplementary Fig. 4. Values are in Supplementary Table 4.

Neutralism Data Fitting and Predictions

Our model of neutralism has only parameters from Monod growth and consumption. Therefore single strain control growths determine all parameters. Initial conditions were chosen with each strain starting at OD600 of 0.04. The simulated result and the experimental result agreed qualitatively. Results are shown in Fig. 2. Values are in Supplementary Table 4.

Cooperation Data Fitting and Predictions

To fit the data for cooperation we grew strains individually with a large amount of added nisin. The added nisin induced the tetracycline immunity genes and allowed us to determine the growth parameters of each strain. In addition both strains were grown without added nisin to determine the killing effectiveness of tetracycline in this system.

The two bacterial strains were then grown together and the parameters describing the cooperative production of nisin were fit from the data. Results are shown in Fig. 3, Supplementary Fig. 5. Values are in Supplementary Table 5.

Competition Data Fitting and Predictions

To fit competition we grew nisin and lcnA producing strains both individually and together and used the resulting experimental data to determine corresponding parameters. Different variants of the green nisin producer and the red lcnA producer were used. A case of competition with the green strain winning is shown in Fig. 3. Additional cases where red wins and marginal cases are shown in Supplementary Fig. 6. Values are in Supplementary Table 5.

Predation Data Fitting and Predictions

The green strain in predation is identical to the green strain in commensalism, but its growth parameters were newly fit with the data from a larger time window of the monoculture experiment of the commensal green strain. This is because predation takes a longer time to occur than commensalism and strain behavior in a longer time window can differ from that in a shorter time. The red strain is grown with nisin to determine its growth parameters. The red strain is also grown without nisin to determine how effective tetracycline is at suppressing its growth.

Co-culture of the strains were used to determine remaining interaction-related parameters. Results are shown in Fig. 3, Supplementary Fig. 7. Values are in Supplementary Table 5.

Initial Conditions for Three- and Four-Strain Ecosystems

All cells in all simulations started out at an initial OD of 0.04.

Spatial Simulation Data and Initial Conditions

Spatial experiments were done by putting a drop containing cells on an agar plate and letting them grow. By volume the cells made up a small fraction of the drop. Simulations were conducted on a 100×100 grid with periodic boundary conditions and homogeneity assumption in each grid to mimic a space equivalent to 1 mm^2 in experiments. To simulate the initial conditions we assumed that the initial cell seeding of each strain follows a Poisson distribution with λ equals to 0.05 multiplied by the relative abundance of the strain. Cell concentration was converted from cell number by multiplying a factor of 0.2. Each simulation was conducted 5 times. Results are shown for one of each simulation. Replicate simulations were used to improve data analysis.

The simulations used parameters from well mixed simulations whenever possible. Effective decay of signaling molecules were assumed faster due to diffusion throughout agar plate. The grid size (Δx) was set as 0.01 mm. The basic time step (Δt) was set as 0.01 h. Additionally, within each basic time step, the reaction terms were integrated with the basic time step while the diffusions were computed via four consecutive smaller time steps (0.0025 h) to improve simulation accuracy.

Results from the simulations are in Supplementary Figs. 8, 9, 11. Values are in Supplementary Table 7.

Entropy

The entropy provides a measure of how much disorder there is in the population of a colony. From the fraction of green cells p_g and red cells p_r in a colony, the entropy S was then calculated as:

$$S = -p_g log_2(p_g) - p_r log_2(p_r) \tag{S41}$$

The entropy was calculated for the experimental data and for the simulations. The simulations were repeated 5 times and the results were averaged. Agreeing with previous work (*15*) cooperation increased entropy while competition decreased entropy.

Results for experimental entropy are in Fig. 6. Results for simulated entropy are in Supplementary Fig. 11.

Intensity Correlation Quotient

Li's Intensity Correlation Quotient (ICQ) (*16*) is a measure of how likely an overdensity(underdensity) of green bacteria is to occur at the same location as an overdensity(underdensity) of red bacteria. It is a normalized and rescaled metric to show colocalization (positive) or exclusion (negative).

$$ICQ = \frac{1}{N^2} \sum_{bins} \theta((g - \bar{g})(r - \bar{r})) - \frac{1}{2} \tag{S42}$$

where N is the number of bins, r and g are the red and green intensity in a bin, $\bar{r}$ and $\bar{g}$ are the average red and green intensity across all bins.

To better see the effects of our engineered circuits, the data was binned into 3x3 bins. The ICQ was calculated over all bins. The theta function is 1 only when both an overdensity of green and red or both an underdensity of green and red occur at the same grid square. Thus positive values indicate colocalization and negative values indicate exclusion.

The ICQ was calculated for both the experimental and simulation data. The simulations were repeated 5 times and the results were averaged. For the simulated data, OD values less than 10^{-1} (i.e., one cell per grid) were rounded down to zero to eliminate remnants from differential equation simulations that is biologically meaningless but affects the ICQ calculation.

Results for experiment are in Fig. 6 and results from simulation are in Supplementary Fig. 11.

Supplementary Note 3: Predictive Modeling of Three- and Four-Strain Ecosystems

Modeling of Three-Strain Ecosystems

The mathematical framework for each three-strain ecosystem was constructed by modularly combining the growth modules for each of the strains, the relevant interaction modules, as well as descriptions of nutrients and signaling molecules. The growth properties of the first and second strain in each ecosystem are those of the two-strain ecosystems, and the third strains are those grown with gusA. The system parameters were unchanged from the two-strain ecosystems except the growth parameters of the third strains that were determined with new experiments. Additionally, as the strains CpBg and AmAg exhibited characteristics distinct from their parents CpB and AmA experimentally, we recalibrated the parameters $d_{S_n,CpBg}$ (0.68) and $d_{S_l,AmAg}$ (1.68) to reflect the differences. Parameter vaules are in Supplementary Tables 4-6. The mathematical models are:

CmA-CmB-CoAg Consortium

$$
\begin{aligned}
\frac{dN_1}{dt} &= g_1(F)N_1 \\
\frac{dN_2}{dt} &= g_2(F)N_2 - I_{t,2}(S_n)N_2 \\
\frac{dN_3}{dt} &= g_3(F)N_3 - I_{t,3}(S_n)N_3 \\
\frac{dS_p}{dt} &= P_{s,3}(S_n,F)N_3 - k_m S_p N_1 - k_p S_p \\
\frac{dS_n}{dt} &= P_{s,1}(S_n,F)N_1 + k_m S_p N_1 - k_n S_n \\
\frac{dF}{dt} &= -\frac{1}{\gamma_1}G_1N_1 - \frac{1}{\gamma_2}G_2N_2 - \frac{1}{\gamma_3}G_3N_3
\end{aligned}
\tag{S43}
$$

CmA-CmB-CoBg Consortium

$$
\begin{aligned}
\frac{dN_1}{dt} &= g_1(F)N_1 \\
\frac{dN_2}{dt} &= g_2(F)N_2 - I_{t,2}(S_n)N_2 \\
\frac{dN_3}{dt} &= g_3(F)N_3 - I_{t,3}(S_n)N_3 \\
\frac{dS_n}{dt} &= P_{s,1}(S_n,F)N_1 - k_n S_n \\
\frac{dF}{dt} &= -\frac{1}{\gamma_1}G_1N_1 - \frac{1}{\gamma_2}G_2N_2 - \frac{1}{\gamma_3}G_3N_3
\end{aligned}
\tag{S44}
$$

AmA-AmB-CpAg Consortium

$$\begin{aligned}
\frac{dN_1}{dt} &= g_1(F)N_1 \\
\frac{dN_2}{dt} &= g_2(F)N_2 - I_{n,2}(S_n)N_2 \\
\frac{dN_3}{dt} &= g_3(F)N_3 \\
\frac{dS_n}{dt} &= P_{s,1}(S_n, F)N_1 + P_{s,3}(S_n, F)N_3 - k_n S_n \\
\frac{dF}{dt} &= -\frac{1}{\gamma_1}G_1N_1 - \frac{1}{\gamma_2}G_2N_2 - \frac{1}{\gamma_3}G_3N_3
\end{aligned} \tag{S45}$$

AmA-AmB-CpBg Consortium

$$\begin{aligned}
\frac{dN_1}{dt} &= g_1(F)N_1 - I_{l,1}(S_l)N_1 \\
\frac{dN_2}{dt} &= g_2(F)N_2 - I_{n,2}(S_n)N_2 - I_{l,2}(S_l)N_2 \\
\frac{dN_3}{dt} &= g_3(F)N_3 - I_{n,3}(S_n)N_3 \\
\frac{dS_n}{dt} &= P_{s,1}(S_n, F)N_1 - k_n S_n \\
\frac{dS_l}{dt} &= r_l N_3 F - k_l S_l \\
\frac{dF}{dt} &= -\frac{1}{\gamma_1}G_1N_1 - \frac{1}{\gamma_2}G_2N_2 - \frac{1}{\gamma_3}G_3N_3
\end{aligned} \tag{S46}$$

CoA-CoB-CmAg Consortium

$$\begin{aligned}
\frac{dN_1}{dt} &= g_1(F)N_1 - I_{t,1}(S_n)N_1 \\
\frac{dN_2}{dt} &= g_2(F)N_2 - I_{t,2}(S_n)N_2 \\
\frac{dN_3}{dt} &= g_3(F)N_3 \\
\frac{dS_p}{dt} &= P_{s,1}(S_n, F)N_1 - k_m S_p(N_2 + N_3) - k_p S_p \\
\frac{dS_n}{dt} &= k_m S_p(N_2 + N_3) + P_{s,3}(S_n, F)N_3 - k_n S_n \\
\frac{dF}{dt} &= -\frac{1}{\gamma_1}G_1N_1 - \frac{1}{\gamma_2}G_2N_2 - \frac{1}{\gamma_3}G_3N_3
\end{aligned} \tag{S47}$$

CoA-CoB-CmBg Consortium

$$\begin{aligned}
\frac{dN_1}{dt} &= g_1(F)N_1 - I_{t,1}(S_n)N_1 \\
\frac{dN_2}{dt} &= g_2(F)N_2 - I_{t,2}(S_n)N_2 \\
\frac{dN_3}{dt} &= g_3(F)N_3 - I_{t,3}(S_n)N_3 \\
\frac{dS_p}{dt} &= P_{s,1}(S_n, F)N_1 - k_m S_p N_2 - k_p S_p \\
\frac{dS_n}{dt} &= k_m S_p N_2 - k_n S_n \\
\frac{dF}{dt} &= -\frac{1}{\gamma_1}G_1N_1 - \frac{1}{\gamma_2}G_2N_2 - \frac{1}{\gamma_3}G_3N_3
\end{aligned} \tag{S48}$$

CpA-CpB-AmAg Consortium

$$\begin{aligned}
\frac{dN_1}{dt} &= g_1(F)N_1 - I_{l,1}(S_l)N_1 \\
\frac{dN_2}{dt} &= g_2(F)N_2 - I_{n,2}(S_n)N_2 \\
\frac{dN_3}{dt} &= g_3(F)N_3 - I_{l,3}(S_l)N_3 \\
\frac{dS_n}{dt} &= P_{s,1}(S_n, F)N_1 + P_{s,3}(S_n, F)N_3 - k_n S_n \\
\frac{dS_l}{dt} &= r_l N_2 F - k_l S_l \\
\frac{dF}{dt} &= -\frac{1}{\gamma_1}G_1N_1 - \frac{1}{\gamma_2}G_2N_2 - \frac{1}{\gamma_3}G_3N_3
\end{aligned} \tag{S49}$$

CpA-CpB-AmBg Consortium

$$\begin{aligned}
\frac{dN_1}{dt} &= g_1(F)N_1 - I_{l,1}(S_l)N_1 \\
\frac{dN_2}{dt} &= g_2(F)N_2 - I_{n,2}(S_n)N_2 \\
\frac{dN_3}{dt} &= g_3(F)N_3 - I_{l,3}(S_l)N_3 - I_{n,3}(S_n)N_3 \\
\frac{dS_n}{dt} &= P_{s,1}(S_n, F)N_1 - k_n S_n \\
\frac{dS_l}{dt} &= r_l N_2 F - k_l S_l \\
\frac{dF}{dt} &= -\frac{1}{\gamma_1}G_1N_1 - \frac{1}{\gamma_2}G_2N_2 - \frac{1}{\gamma_3}G_3N_3
\end{aligned} \tag{S50}$$

Similar to the two-strain cases, nutrient reductions (G_1, G_2, G_3) for the above eight consortia are g_1, $g_2 - \varepsilon_t I_{t,2}$ and $g_3 - \varepsilon_t I_{t,3}$ for Eq. S43, g_1, $g_2 - \varepsilon_t I_{t,2}$ and $g_3 - \varepsilon_t I_{t,3}$ for Eq. S44, g_1, $g_2 - \varepsilon_n I_{n,2}$ and g_3 for Eq. S45, $g_1 - \varepsilon_l I_{l,1}$, $g_2 - \varepsilon_n I_{n,2} - \varepsilon_l I_{l,2}$ and $g_3 - \varepsilon_n I_{n,3}$ for Eq. S46, $g_1 - \varepsilon_t I_{t,1}$, $g_2 - \varepsilon_t I_{t,2}$ and g_3 for Eq. S47, $g_1 - \varepsilon_t I_{t,1}$, $g_2 - \varepsilon_t I_{t,2}$ and $g_3 - \varepsilon_t I_{t,3}$ for Eq. S48, $g_1 - \varepsilon_l I_{l,1}$, $g_2 - \varepsilon_n I_{n,2}$ and $g_3 - \varepsilon_l I_{l,3}$ for Eq. S49, $g_1 - \varepsilon_l I_{l,1}$, $g_2 - \varepsilon_n I_{n,2}$ and $g_3 - \varepsilon_l I_{l,3} - \varepsilon_n I_{n,3}$ for Eq. S50, respectively.

Modeling of Four-Strain Ecosystems

The mathematical framework for each four-strain ecosystem was constructed by modularly combining the growth modules for each of the strains, the relevant interaction modules, as well as descriptions of nutrients and signaling molecules. The growth properties of the first and second strain in each ecosystem are those of the two-strain ecosystems, the third strains are those grown with gusA, and the fourth strains are those lacking reporters. The system parameters were unchanged from the two- and three-strain ecosystems and only the growth parameters of the fourth strains were newly determined with experiment. Parameter values are in Supplementary Tables 4-6. The mathematical models are:

CoA-CoB-CmAg-PrBn Consortium

$$\begin{aligned}
\frac{dN_1}{dt} &= g_1(F)N_1 - I_{t,1}(S_n)N_1 - I_{l,1}(S_l)N_1 \\
\frac{dN_2}{dt} &= g_2(F)N_2 - I_{t,2}(S_n)N_2 - I_{l,2}(S_l)N_2 \\
\frac{dN_3}{dt} &= g_3(F)N_3 - I_{l,3}(S_l)N_3 \\
\frac{dN_4}{dt} &= g_4(F)N_4 - I_{t,4}(S_n)N_4 \\
\frac{dS_p}{dt} &= P_{s,1}(S_n, F)N_1 - k_m S_p(N_2 + N_3) - k_p S_p \\
\frac{dS_n}{dt} &= k_m S_p(N_2 + N_3) + P_{s,3}(S_n, F)N_3 - k_n S_n \\
\frac{dS_l}{dt} &= r_l N_4 F - k_l S_l \\
\frac{dF}{dt} &= -\frac{1}{\gamma_1}G_1N_1 - \frac{1}{\gamma_2}G_2N_2 - \frac{1}{\gamma_3}G_3N_3 - \frac{1}{\gamma_4}G_4N_4
\end{aligned} \tag{S51}$$

CoA-CoB-CmAg-CmBn Consortium

$$\begin{aligned}
\frac{dN_1}{dt} &= g_1(F)N_1 - I_{t,1}(S_n)N_1 \\
\frac{dN_2}{dt} &= g_2(F)N_2 - I_{t,2}(S_n)N_2 \\
\frac{dN_3}{dt} &= g_3(F)N_3 \\
\frac{dN_4}{dt} &= g_4(F)N_4 - I_{t,4}(S_n)N_4 \\
\frac{dS_p}{dt} &= P_{s,1}(S_n, F)N_1 - k_m S_p(N_2 + N_3) - k_p S_p \\
\frac{dS_n}{dt} &= k_m S_p(N_2 + N_3) + P_{s,3}(S_n, F)N_3 - k_n S_n \\
\frac{dF}{dt} &= -\frac{1}{\gamma_1}G_1N_1 - \frac{1}{\gamma_2}G_2N_2 - \frac{1}{\gamma_3}G_3N_3 - \frac{1}{\gamma_4}G_4N_4
\end{aligned} \tag{S52}$$

CmA-PrB-CoAg-CmBn Consortium

$$\begin{aligned}
\frac{dN_1}{dt} &= g_1(F)N_1 - I_{l,1}(S_l)N_1 \\
\frac{dN_2}{dt} &= g_2(F)N_2 - I_{t,2}(S_n)N_2 \\
\frac{dN_3}{dt} &= g_3(F)N_3 - I_{t,3}(S_n)N_3 - I_{l,3}(S_l)N_3 \\
\frac{dN_4}{dt} &= g_4(F)N_4 - I_{t,4}(S_n)N_4 - I_{l,4}(S_l)N_4 \\
\frac{dS_p}{dt} &= P_{s,3}(S_n, F)N_3 - k_m S_p N_1 - k_p S_p \\
\frac{dS_n}{dt} &= P_{s,1}(S_n, F)N_1 + k_m S_p N_1 - k_n S_n \\
\frac{dS_l}{dt} &= r_l N_2 F - k_l S_l \\
\frac{dF}{dt} &= -\frac{1}{\gamma_1}G_1 N_1 - \frac{1}{\gamma_2}G_2 N_2 - \frac{1}{\gamma_3}G_3 N_3 - \frac{1}{\gamma_4}G_4 N_4
\end{aligned} \tag{S53}$$

CpA-CpB-AmAg-AmBn Consortium

$$\begin{aligned}
\frac{dN_1}{dt} &= g_1(F)N_1 - I_{l,1}(S_l)N_1 \\
\frac{dN_2}{dt} &= g_2(F)N_2 - I_{n,2}(S_n)N_2 \\
\frac{dN_3}{dt} &= g_3(F)N_3 - I_{l,3}(S_l)N_3 \\
\frac{dN_4}{dt} &= g_4(F)N_4 - I_{l,4}(S_l)N_4 - I_{n,4}(S_n)N_4 \\
\frac{dS_n}{dt} &= P_{s,1}(S_n, F)N_1 + P_{s,3}(S_n, F)N_3 - k_n S_n \\
\frac{dS_l}{dt} &= r_l N_2 F - k_l S_l \\
\frac{dF}{dt} &= -\frac{1}{\gamma_1}G_1 N_1 - \frac{1}{\gamma_2}G_2 N_2 - \frac{1}{\gamma_3}G_3 N_3 - \frac{1}{\gamma_4}G_4 N_4
\end{aligned} \tag{S54}$$

Notably, nutrient reductions (G_1-G_4) in the above systems are $g_1 - \varepsilon_t I_{t,1} - \varepsilon_l I_{l,1}$, $g_2 - \varepsilon_t I_{t,2} - \varepsilon_l I_{l,2}$, $g_3 - \varepsilon_l I_{l,3}$ and $g_4 - \varepsilon_t I_{t,4}$ for Eq. S51; $g_1 - \varepsilon_t I_{t,1}$, $g_2 - \varepsilon_t I_{t,2}$, g_3 and $g_4 - \varepsilon_t I_{t,4}$ for Eq. S52; $g_1 - \varepsilon_l I_{l,1}$, $g_2 - \varepsilon_t I_{t,2}$, $g_3 - \varepsilon_t I_{t,3} - \varepsilon_l I_{l,3}$ and $g_4 - \varepsilon_t I_{t,4} - \varepsilon_l I_{l,4}$ for Eq. S53; $g_1 - \varepsilon_l I_{l,1}$, $g_2 - \varepsilon_n I_{n,2}$, $g_3 - \varepsilon_l I_{l,3}$ and $g_4 - \varepsilon_l I_{l,4} - \varepsilon_n I_{n,4}$ for Eq. S54, respectively.

Supplementary Note 4: Spatial Ecosystem Modeling

General Modeling Framework

In addition to modeling well mixed interactions we also modeled the spatial effect of our engineering circuits in a viscous environment. For spatial simulations we took the well mixed models and changed them to describe an environment where the spatial distribution of bacteria, nutrients and signaling molecules matters. To incorporate spatial effect we made bacteria concentration, nutrient concentration, and signaling molecule concentration depend on both space and time. To travel between locations we also need to introduce diffusion. Taking the general equation Eq. $S1$ and adding spatial dependence and diffusion we arrive at a general spatial simulation form:

$$
\begin{aligned}
\frac{\partial N_1}{\partial t} &= D\nabla^2 N_1 + g_1(F)N_1 - I_1(S_1,S_2)N_1 \\
\frac{\partial N_2}{\partial t} &= D\nabla^2 N_2 + g_2(F)N_2 - I_2(S_1,S_2)N_2 \\
\frac{\partial S_1}{\partial t} &= D_{s1}\nabla^2 S_1 + P_1(S_1,S_2,N_1,N_2,F) - k_1 S_1 \\
\frac{\partial S_2}{\partial t} &= D_{s2}\nabla^2 S_2 + P_2(S_1,S_2,N_1,N_2,F) - k_2 S_2 \\
\frac{\partial F}{\partial t} &= D_f\nabla^2 F - \frac{1}{\gamma_1}G_1(F,S_1,S_2)N_1 - \frac{1}{\gamma_2}G_2(F,S_1,S_2)N_2
\end{aligned}
\tag{S55}
$$

With D, D_{s1}, D_{s2}, and D_f being the diffusion coefficients of cells, signaling molecules 1 and 2, and nutrient.

Diffusion

To model diffusion we discretized the diffusion terms of Eq. S55 in a form of $X_{ij}(t+\Delta t) = X_{ij}(t) + \frac{\Delta t}{(\Delta x)^2}\sum_{i'j'}\left(D_{i'j'\to ij}X_{i'j'}(t) - D_{ij\to i'j'}X_{ij}(t)\right)$, where X_{ij} is the density of cells, signaling molecules or nutrient at grid (i,j), t is time, Δt is time step, Δx is grid size, $D_{i'j'\to ij}$ and $D_{ij\to i'j'}$ are diffusion coefficients of X from the grid (i',j') to (i,j) and grid (i,j) to (i',j'), $\sum_{i'j'}$ is the summation over the grids adjacent to (i,j). In our simulations, the diffusion coefficients take the unit of mm^2/h.

The diffusion coefficients D_{s1}, D_{s1} and D_f are constants but D is not. This is because, owing to volume exclusion of cellular physical sizes, increasing cell density of a grid increases the likelihood of cells to diffuse out the grid and hinders cells in adjacent grids from diffusing in. Thus, cellular diffusion coefficient was modeled as

$$
D_{ij\to i'j'} = D_0\left[\frac{(N_{1,ij}+N_{2,ij})^{\theta_o}}{(N_{to})^{\theta_o}+(N_{1,ij}+N_{2,ij})^{\theta_o}}\right]\left[\frac{(N_{ti})^{\theta_i}}{(N_{ti})^{\theta_i}+(N_{1,i'j'}+N_{2,i'j'})^{\theta_i}}\right] \tag{S56}
$$

where D_0 is the maximal diffusion constant, N_{to} and N_{ti} are the Hill function thresholds, and θ_o and θ_i are the Hill coefficients. $N_{1,ij}$, $N_{2,ij}$ $N_{1,i'j'}$ and $N_{2,i'j'}$ are the densities of strains 1 and 2 in the grids (i,j) and (i',j') respectively. In the simulations, we chose $N_{to} = 3.0$, $N_{ti} = 1.0$, $\theta_o = \theta_i = 4$.

Neutralism

Following the general framework for spatial simulation Eq. $S55$, the spatial model of neutralism is:

$$
\begin{aligned}
\frac{\partial N_1}{\partial t} &= D\nabla^2 N_1 + g_1(F)N_1 \\
\frac{\partial N_2}{\partial t} &= D\nabla^2 N_2 + g_2(F)N_2 \\
\frac{\partial F}{\partial t} &= D_f\nabla^2 F - \frac{1}{\gamma_1}G_1N_1 - \frac{1}{\gamma_2}G_2N_2
\end{aligned} \tag{S57}
$$

Cooperation

Following the general framework for spatial simulation Eq. $S55$, the spatial model of cooperation is:

$$
\begin{aligned}
\frac{\partial N_1}{\partial t} &= D\nabla^2 N_1 + g_1(F)N_1 - I_{t,1}(S_n)N_1 \\
\frac{\partial N_2}{\partial t} &= D\nabla^2 N_2 + g_2(F)N_2 - I_{t,2}(S_n)N_2 \\
\frac{\partial S_p}{\partial t} &= D_p\nabla^2 S_p + P_s(S_n, F)N_1 - k_mS_pN_2 - k_pS_p \\
\frac{\partial S_n}{\partial t} &= D_n\nabla^2 S_n + k_mS_pN_2 - k_nS_n \\
\frac{\partial F}{\partial t} &= D_f\nabla^2 F - \frac{1}{\gamma_1}G_1N_1 - \frac{1}{\gamma_2}G_2N_2
\end{aligned} \tag{S58}
$$

Competition

Following the general framework for spatial simulation Eq. $S55$, the spatial model of competition is:

$$
\begin{aligned}
\frac{\partial N_1}{\partial t} &= D\nabla^2 N_1 + g_1(F)N_1 - I_l(S_l)N_1 \\
\frac{\partial N_2}{\partial t} &= D\nabla^2 N_2 + g_2(F)N_2 - I_n(S_n)N_2 \\
\frac{\partial S_n}{\partial t} &= D_n\nabla^2 S_n + P_s(S_n, F)N_1 - k_nS_n \\
\frac{\partial S_l}{\partial t} &= D_l\nabla^2 S_l + r_lN_2F - k_lS_l \\
\frac{\partial F}{\partial t} &= D_f\nabla^2 F - \frac{1}{\gamma_1}G_1N_1 - \frac{1}{\gamma_2}G_2N_2
\end{aligned} \tag{S59}
$$

References

1. Kuipers O.P., de Ruyter P.G., Kleerebezem M. and de Vos W.M. Quorum sensing-controlled gene expression in lactic acid bacteria, *J. Biotechnol.* **64**, 15-21 (1998).

2. Le Loir, Y., Gruss A., Ehrlich S.D. and Langella P. A nine-residue synthetic propeptide enhances secretion efficiency of heterologous proteins in lactococcus lactis, *J. Bacteriol.* **180**, 1895-1903 (1998).

3. Kong W., Kapuganti V.S. and Lu T. A gene network engineering platform for lactic acid bacteria, *Nucleic Acids Res.* **44**, e37 (2016).

4. Kong W. and Lu T. Cloning and optimization of a nisin biosynthesis pathway for bacteriocin harvest, *ACS Synth. Biol.* **3**, 439-445 (2014).

5. Mierau I. and Kleerebezem M. 10 years of the nisin-controlled gene expression system (NICE) in lactococcus lactis, *Appl. Microbiol. Biotechnol.* **68**, 705-717 (2005).

6. Kuipers O.P., Beerthuyzen M.M., de Ruyter P.G., Luesink E.J. and de Vos W.M. Autoregulation of nisin biosynthesis in lactococcus lactis by signal transduction, *J. Biol. Chem.* **270**, 27299-27304 (1995).

7. Mierau I., Leij P., van Swam I., Blommestein B., Floris E., Mond J. and Smid E.J. Industrial-scale production and purification of a heterologous protein in lactococcus lactis using the nisin-controlled gene expression system NICE: the case of lysostaphin, *Microb. Cell Fact.* **4**, 15 (2005).

8. Delves-Broughton J., Blackburn P., Evans R.J. and Hugenholtz J. Applications of the bacteriocin, nisin, *Antonie van Leeuwenhoek* **69**, 193-202 (1996).

9. Hassan M., Kjos M., Nes I.F., Diep D.B. and Lotfipour F. Natural antimicrobial peptides from bacteria: characteristics and potential applications to fight against antibiotic resistance, *J. Appl. Microbiol.* **113**, 723-736 (2012).

10. Landersdorfer C.B., Ly N.S., Xu H., Tsuji B.T. and Bulitta J.B. Quantifying subpopulation synergy for antibiotic combinations via mechanism-based modeling and a sequential dosing design, *Antimicrob. Agents Chemother.* **57**, 2343-2351 (2013).

11. Regoes R.R., Wiuff C., Zappala R.M., Kim N., Baquero F., Levin B.R. and Garner K.N. Pharmacodynamic functions: a multiparameter approach to the design of antibiotic treatment regimens pharmacodynamic functions, *Antimicrob. Agents Chemother.* **48**, 3670-3676 (2004).

12. Holo H., Nilssen O. and Nes I.F. Lactococcin A, a new bacteriocin from lactococcus lactis subsp. cremoris: Isolation and characterization of the protein and its gene, *J. Bacteriol.* **173**, 3879-3887 (1991).

13. Smith H.L., Howland M.C., Szmodis A.W., Li Q., Daemen L.L., Parikh A.N. and Majewski J. Early stages of oxidative stress-induced membrane permeabilization: a neutron reflectometry study, *J. Am. Chem. Soc.* **131** 3631-3638 (2009).

14. Cheigh C.I. and Pyun Y.R. Nisin biosynthesis and its properties, *Biotechnol. Lett.* **27**, 1641-1648 (2005).

15. Blanchard A.E. and Lu, T. Bacterial social interactions drive the emergence of differential spatial colony structures, *BMC Syst. Biol.* **9**, 59 (2015).

16. Li Q., Lau A., Morris T.J., Guo L., Fordyce C.B. and Stanley E.F.. A syntaxin 1, Galpha(o), and N-type calcium channel complex at a presynaptic nerve terminal: analysis by quantitative immunocolocalization *J. Neurosci.* **24**, 4070-4081 (2004).
